# Separating hydrogen and oxygen evolution in alkaline water electrolysis using nickel hydroxide

**DOI:** 10.1038/ncomms11741

**Published:** 2016-05-20

**Authors:** Long Chen, Xiaoli Dong, Yonggang Wang, Yongyao Xia

**Affiliations:** 1 Department of Chemistry, Shanghai Key Laboratory of Molecular Catalysis and Innovative Materials, Institute of New Energy, iChEM (Collaborative Innovation Center of Chemistry for Energy Materials), Fudan University, Shanghai 200433, China

## Abstract

Low-cost alkaline water electrolysis has been considered a sustainable approach to producing hydrogen using renewable energy inputs, but preventing hydrogen/oxygen mixing and efficiently using the instable renewable energy are challenging. Here, using nickel hydroxide as a redox mediator, we decouple the hydrogen and oxygen production in alkaline water electrolysis, which overcomes the gas-mixing issue and may increase the use of renewable energy. In this architecture, the hydrogen production occurs at the cathode by water reduction, and the anodic Ni(OH)_2_ is simultaneously oxidized into NiOOH. The subsequent oxygen production involves a cathodic NiOOH reduction (NiOOH→Ni(OH)_2_) and an anodic OH^−^ oxidization. Alternatively, the NiOOH formed during hydrogen production can be coupled with a zinc anode to form a NiOOH-Zn battery, and its discharge product (that is, Ni(OH)_2_) can be used to produce hydrogen again. This architecture brings a potential solution to facilitate renewables-to-hydrogen conversion.

## Abstract (web summary)

Alkaline water electrolyzers are promising devices for hydrogen generation, although hydrogen/oxygen mixing can be problematic. Here, the authors use nickel hydroxide as a redox mediator to decouple the hydrogen and oxygen production, overcoming the gas-mixing issue.

## Graphical abstract

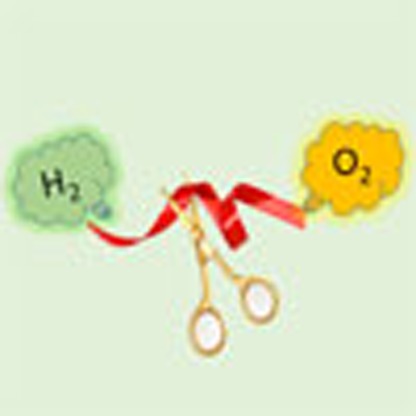

Hydrogen has long been considered a promising alternative to unsustainable fossil fuels because it is vital for the production of commodity chemicals such as ammonia and has great potential as a clean-burning fuel^1^,^2^. However, 90% of the world's hydrogen is currently obtained by the reformation of fossil fuels^3^,^4^, which consumes much energy and is accompanied by serious CO_2_ emissions. To realize a hydrogen-based economy, hydrogen must be efficiently and sustainably produced^1^,^2^,^3^,^4^,^5^,^6^,^7^,^8^. Accordingly, the state-of-the-art (photo) electrocatalysts for water splitting are attracting extensive attention^9^,^10^,^11^,^12^,^13^. Advanced water electrolysis has been considered as one of the most efficient and reliable approaches to producing hydrogen from renewable energy, such as solar, wind and hydropower, for grid-scale energy storage^14^,^15^,^16^ because the electrolysis of water at room temperature stands out as a scalable technology, for which the only required inputs are water and energy (in the form of electricity)^17^. Therefore, electrochemical water splitting is attracting extensive attention^18^,^19^,^20^,^21^,^22^,^23^,^24^,^25^,^26^,^27^,^28^,^29^,^30^,^31^. However, the general application of water electrolysis currently faces a great challenge.

Room-temperature water electrolysis is generally performed under acidic or alkaline conditions. The water electrolysis under acidic condition is performed in an electrolyser with a proton exchange membrane (PEM); thus, it is called PEM water electrolysis^32^,^33^,^34^,^35^. Although PEM water electrolysis systems offer several advantages, such as high energy efficiency, a great hydrogen production rate and a compact design, their application remains hampered by the high cost of the catalysts and membranes^36^,^37^. The acidic environment limits the catalysts for the oxygen-evolving reaction (OER) and hydrogen-evolving reaction (HER) to noble metals. The expensive PEM, which is necessary to prevent the H_2_/O_2_ mixing, also significantly increases the cost. Furthermore, the short durability of the membrane makes PEM electrolysers too expensive for general applications^37^,^38^. Cronin's group recently developed a new method to split the conventional PEM water electrolysis process into two steps using the polyoxometalate H_3_PMo_12_O_40_ as a buffer for redox equivalents^39^,^40^. The separate generation of O_2_ and H_2_ simplifies gas handling, puts less stringent demands on the membrane of PEM water electrolysis, and potentially reduces cost^41^. Cronin *et al*. undoubtedly provided an interesting and bright idea for future water electrolysis technology. However, their results^39^,^40^ only focus on PEM water electrolysis (acid condition) and cannot be used in alkaline water electrolysis.

Compared with PEM water electrolysis, alkaline water electrolysis exhibits inherent low-cost characteristic because it can use a non-precious catalyst and a porous separator^36^,^37^. However, alkaline water electrolysers are difficult to shut down/start up, and their output cannot be quickly ramped because the pressures on the anode and cathode sides of the cell must always be equalized to prevent gas crossover through the porous cell separator^36^. Therefore, it is notably difficult to efficiently use the intermittent and fluctuating power from renewable energy for conventional alkaline water electrolysis. As an alternative to the porous separator, alkaline anion exchange membranes are considered suitable candidate materials that can easily prevent the gas mixing in alkaline water electrolysers^36^,^42^. However, similar to PEM water electrolysis, the cost and short durability of alkaline anion exchange membranes limit the scalable application. In particular, high-pressure gases in the electrolytic cell aggravate the membrane degradation^40^. Thus, to make renewables-to-hydrogen conversion both practically and economically more attractive, new electrolyser systems must be developed to prevent product gases from mixing over a range of current densities and effectively use the low-cost characteristic of alkaline water electrolysis. It should be a promising solution to separate H_2_ production and O_2_ production. However, the method to separate conventional alkaline water electrolysis into two steps has never been reported.

In the report by Cronin *et al*.^39^, a polyoxometalate-based redox mediator was used as an electron-coupled-proton buffer in an acid solution to decouple the H_2_ and O_2_ evolution in PEM water electrolysis. Similarly, it should be a precondition to find a stable electron-coupled-proton buffer in an alkaline solution to separate the H_2_ and O_2_ evolution in alkaline water electrolysis. Nickel hydroxide has been widely used in rechargeable batteries using alkaline electrolytes^43^. Its charge and discharge depend on the reversible transformation of Ni(OH)_2_/NiOOH^44^,^45^,^46^,^47^, which can be explained as an electron-coupled proton release/storage process (that is, H^+^ de-intercalation from Ni(OH)_2_ or intercalation into NiOOH, Supplementary Fig. 1). Therefore, it should be an interesting topic to use nickel hydroxide as an electron-coupled-proton buffer to separate the H_2_ and O_2_ evolution in alkaline water electrolysis.

Here, using nickel hydroxide (Ni(OH)_2_/NiOOH) as a redox mediator, we successfully split the conventional alkaline water electrolysis process into separate H_2_ and O_2_ production steps, which well overcomes the gas-mixing issue and increases the use of renewable energy. In this architecture, the H_2_ production involves the cathodic H_2_O reduction and anodic Ni(OH)_2_ oxidization (Ni(OH)_2_→NiOOH), and the subsequent O_2_ production depends on the cathodic NiOOH reduction and anodic OH^−^ oxidization. In addition, the formed NiOOH during the H_2_ production can be coupled with a zinc anode to form a NiOOH-Zn battery, which provides an interesting rechargeable system that produces H_2_ during the electrolysis and delivers energy on the discharge of the NiOOH-Zn battery.

## Results

### Mechanism of the two-step alkaline water electrolysis

**Figure 1:**
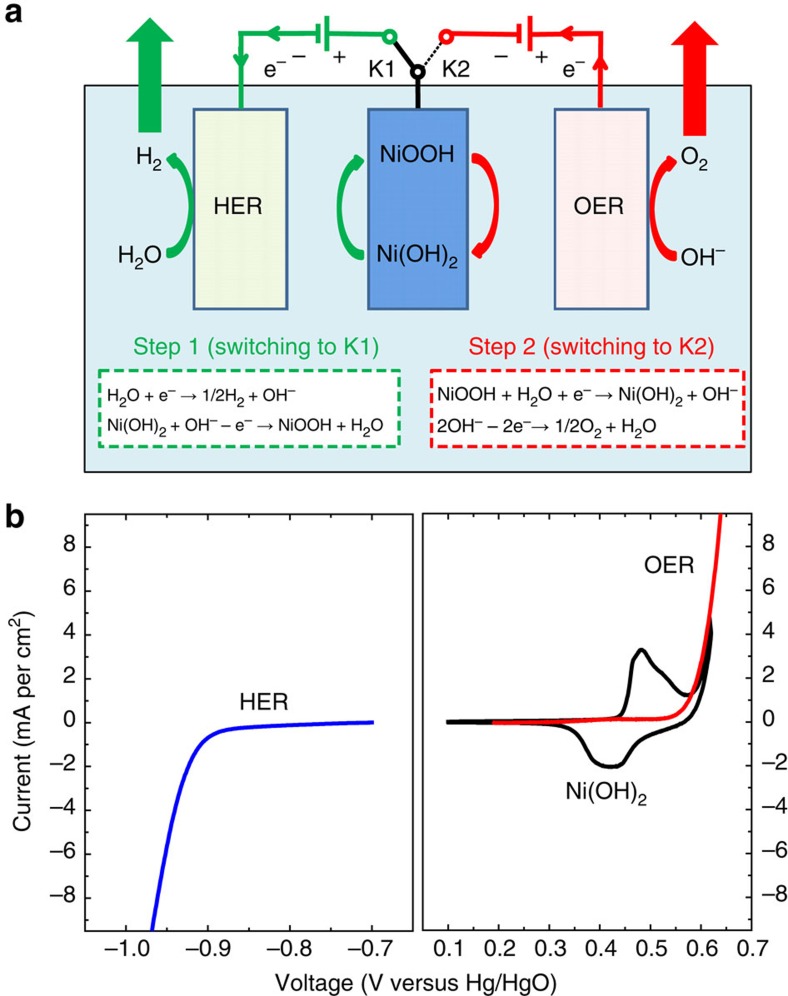
Mechanism of the two-step alkaline water electrolysis. (**a**) A schematic of the operation mechanism of the cell, where Step 1 (H_2_ production; switching to K1) involves the cathodic reduction of H_2_O on the HER electrode (H_2_O+e^−^→1/2H_2_+OH^−^) and anodic oxidization in the Ni(OH)_2_ electrode (Ni(OH)_2_+OH^−^—e^−^→ NiOOH+H_2_O). Step 2 (O_2_ production; switching to K2) includes the cathodic reduction of the NiOOH electrode (NiOOH+H_2_O+e^−^→Ni(OH)_2_+OH^−^) and anodic OH^−^ oxidization on the OER electrode (2OH^−^—2e^−^→ 1/2O_2_+H_2_O). (**b**) CV curve of the Ni(OH)_2_ film electrode at a scan rate of 5 mV s^−1^ in 1 M KOH (black line), the linear sweep voltammetric (LSV) data of the commercial RuO_2_/IrO_2_-coated Ti-mesh electrode for the OER at a scan rate of 5 mV s^−1^ in 1 M KOH (red line) and the LSV data of the commercial Pt-coated Ti-mesh electrode for the HER in 1 M KOH with a sweep rate of 5 mV s^−1^ (blue line).

As shown in Fig. 1a, the H_2_ production (Step 1) involves a cathodic reduction of H_2_O on the HER electrode (H_2_O→H_2_) and a simultaneous anodic oxidization of the Ni(OH)_2_ electrode (Ni(OH)_2_→ NiOOH). The subsequent O_2_ production (Step 2) occurs on the OER electrode by an anodic oxidization of OH^−^ (OH^−^→ O_2_), whereas the NiOOH cathode is reduced to Ni(OH)_2_. This approach leads to a device architecture for the alkaline electrolytic cell with several important advantages. First, the separate generation of O_2_ and H_2_ prevents the product gases from mixing over a range of current densities and simplifies the gas handling, which greatly increase the operation flexibility of alkaline electrolytic cells and make them suitable to be driven by sustainable energy (such as solar energy). Second, this device architecture can produce highly pure H_2_ and O_2_ with no membrane, which further reduces the cost of the alkaline water electrolysis technology. Third, the separate H_2_ and O_2_ productions require different driving voltages (or power inputs), which implies that we can flexibly use sustainable energy (such as solar or wind power) for H_2_ production or O_2_ production based on the output variation in these unstable power sources. Finally, the NiOOH that forms during the H_2_ production (that is, Step 1) can be coupled with a zinc anode to form a NiOOH-Zn battery for energy storage, and its discharge depends on the cathodic reduction of the NiOOH electrode (NiOOH→ Ni(OH)_2_) and the anodic oxidization of the zinc electrode (Zn→ ZnO_2_^2−^). Herein, it should be noted that the cathodic reduction potential of NiOOH (0.45 V versus Hg/HgO) is significantly higher than the anodic oxidization potential of zinc (−1.15 V versus Hg/HgO) (ref. 48). Therefore, the NiOOH cathode and Zn anode can be coupled to form the NiOOH-Zn battery system that has been commercialized^49^,^50^,^51^.) Its discharge product (Ni(OH)_2_) can be used to produce H_2_ again, which provides an interesting rechargeable cycle that produces H_2_ with the charge (that is, electrolysis in Step 1) and delivers energy with the discharge of the NiOOH-Zn battery.

In this work, nickel hydroxide, which is the conventional electrode material for commercial rechargeable Ni-MH or Ni-Cd batteries, was used as a redox mediator to split the conventional alkaline water electrolysis process into two steps. Before the fabrication of this alkaline water electrolytic cell, the electrochemical profile of Ni(OH)_2_ in an alkaline electrolyte (1 M KOH) was investigated using a cyclic voltammogram (CV) with a typical three-electrode system, which used a Pt plate and a Hg/HgO electrode as the counter and reference electrodes, respectively. Carbon-nanotube-supported Ni(OH)_2_ particles were used as the active material to prepare the Ni(OH)_2_-based film electrode (see the Methods and Supplementary Fig. 2 for details) for the CV measurement, where the carbon nanotube support with high electronic conductivity was only used to alleviate the polarization that arose from the electrode impedance. The CV curve of Ni(OH)_2_ at a scan rate of 5 mV s^−1^ is shown in Fig. 1b (black line). The OER and HER potentials of the commercial RuO_2_/IrO_2_-coated Ti-mesh electrode and Pt-coated Ti-mesh electrode were also investigated using the three-electrode method for comparison (see the red and blue lines in Fig. 1b). As shown in Fig. 1b, a couple of redox peaks are clearly observed at 0.43 and 0.49 V (versus Hg/HgO) in the CV curve of the Ni(OH)_2_ electrode because of the reversible cycling between Ni(OH)_2_ and NiOOH. Obviously, the special potential window for the Ni(OH)_2_/NiOOH redox couple is located between the onset potential for the OER and the onset potential for the HER. The result indicates that Ni(OH)_2_ can be used as a redox mediator to split the conventional alkaline water electrolysis process into two steps according to Fig. 1a. The galvanostatic charge-discharge curve of the Ni(OH)_2_ electrode at a current density of 0.2 A g^−1^ is shown in Supplementary Fig. 3 to clarify the specific capacity of Ni(OH)_2_ (see the corresponding discussion about Supplementary Fig. 3).

### Performance of the two-step alkaline water electrolysis

**Figure 2:**
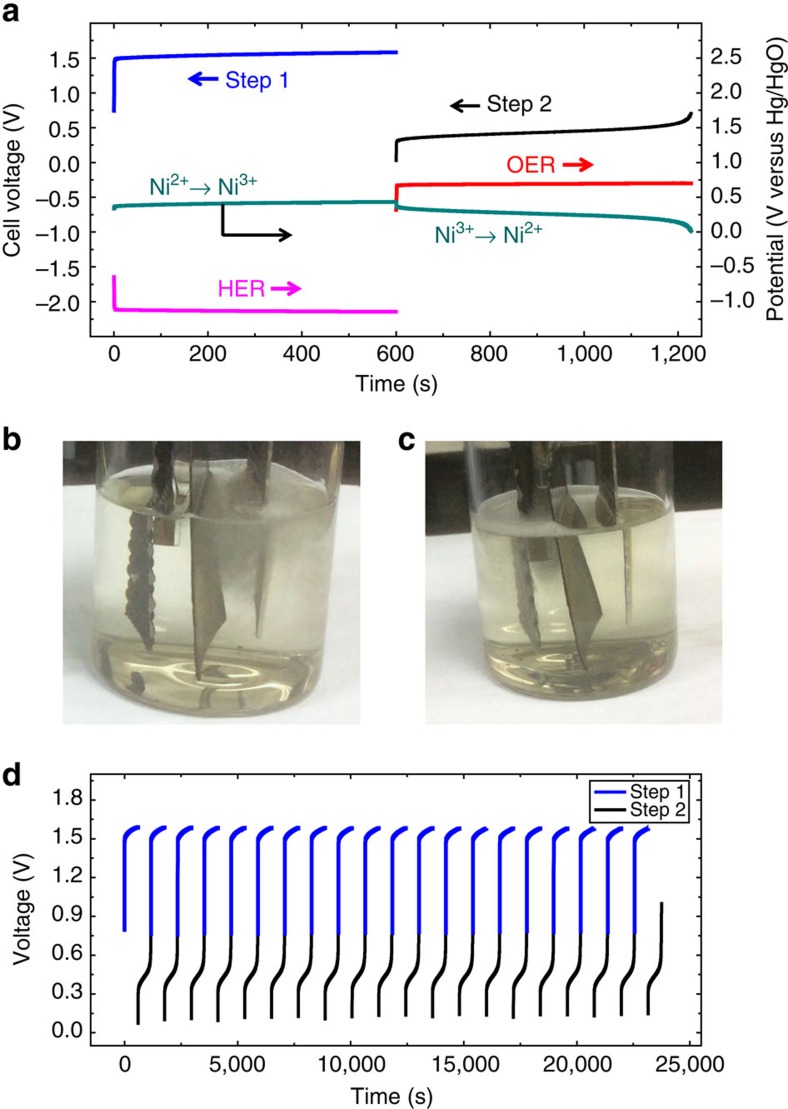
Performance of the two-step alkaline water electrolysis. (**a**) Chronopotentiometry curve (cell voltage versus time) of the cell at a constant applied current of 200 mA, where the voltages for H_2_ production (Step 1) and O_2_ production (Step 2) are labelled by the blue and black lines, respectively. Chronopotentiometry data (potential versus time) of the HER electrode (pink line), Ni(OH)_2_ electrode (green line) and OER electrode (red line) are provided. ((Voltage of Step 1)=(Potential of Ni^2+^→Ni^3+^)—(Potential of HER); (Voltage of Step 2)=(Potential of OER)—(Potential of Ni^3+^→Ni^2+^)). (**b**,**c**) Photo profiles of the H_2_/O_2_ generation in Steps 1 and 2, where it can be detected that H_2_ and O_2_ are produced on the HER (**b**) and OER electrodes (**c**), respectively, at separate times (Supplementary Movies 1 and 2 further confirm this point). (**d**) Chronopotentiometry curve (cell voltage versus time) of the H_2_/O_2_ generation cycle with an applied current of 200 mA and a step time of 600 s, where the chronopotentiometry data of Step 1 (H_2_ generation) and Step 2 (O_2_ generation) are labelled with the blue and black lines, respectively.

To test the hypothesis in Fig. 1a, an alkaline water electrolytic cell was constructed with a commercial Pt-coated Ti-mesh electrode (Supplementary Fig. 4) for the HER, a commercial RuO_2_/IrO_2_-coated Ti-mesh electrode for the OER (Supplementary Fig. 5) and a commercial Ni(OH)_2_ electrode of conventional Ni-MH or Ni-Cd batteries (Supplementary Fig. 6). The photo profile of the cell is shown in Supplementary Fig. 7, which shows that the Ni(OH)_2_ electrode (2.5 × 4 cm^2^) is located between the HER electrode (2.5 × 4 cm^2^) and the OER electrode (2.5 × 4 cm^2^). The water electrolysis of the cell was investigated by chronopotentiometry measurements with different applied currents of 100–500 mA. The chronopotentiometry curve (cell voltage versus time) of the electrolytic cell at a constant applied current of 200 mA is shown in Fig. 2a. The chronopotentiometry data of the anode (anodic potential versus time) and cathode (cathodic potential versus time) were also investigated during the electrolysis process and are provided in Fig. 2a. The electrolysis process includes two steps (Steps 1 and 2) with different cell voltages. As shown in Fig. 2a, Step 1 (that is, the H_2_-production process) exhibits a cell voltage of ∼1.6 V, which arises from the difference between the anodic potential of 0.5 V (versus Hg/HgO) of the Ni(OH)_2_ oxidation (Ni(OH)_2_→NiOOH) and the cathodic potential of −1.1 V (versus Hg/HgO) of the H_2_O reduction (H_2_O→H_2_). In Step 2 (that is, the O_2_-production process), the cell voltage is 0.4 V, which is equal to the potential difference (0.7–0.3 V versus Hg/HgO) between the anodic oxidation of OH^−^ (OH^−^→O_2_) and the cathodic reduction of NiOOH (NiOOH→ Ni(OH)_2_). In Step 2, the cell voltage sharply increases sharply at the end of electrolysis (Fig. 2a), which indicates that all of the NiOOH has been reduced to Ni(OH)_2_. In other words, the electrolysis in Step 2 automatically finished after 600 s (=1,200–600 s), which is equal to the electrolysis time in Step 1 at the identical current of 200 mA. The equal electrolysis time clearly indicates a Coulombic efficiency of ∼100%. Photo profiles of the H_2_ generation in Step 1 and O_2_ generation in Step 2 are shown in Fig. 2b,c to further characterize the separated steps. In addition, the video evidence also clearly demonstrates the separate H_2_/O_2_ generation directly (Supplementary Movies 1 and 2). To clarify the operation flexibility of this electrolyser, the water electrolysis was also investigated at a lower current of 100 mA and a higher current of 500 mA (Supplementary Fig. 8). It should be noted that as a notably mature electrode material, Ni(OH)_2_ exhibits high efficiency and a long cycle life. These characteristics are also notably important to facilitate the cycle of H_2_ generation (Step 1) and O_2_ generation (Step 2). To demonstrate this point, the H_2_/O_2_ generation cycle performance was investigated with an applied current of 200 mA. As shown in Fig. 2d, this alkaline electrolytic cell exhibits stable H_2_ and O_2_ generation over 20 consecutive cycles. Furthermore, 100 consecutive cycles of H_2_/O_2_ generation are shown in Supplementary Fig. 9 to further demonstrate the stability. As shown in Fig. 2d (or Fig. 2a), the separate H_2_ production (Step 1) and O_2_ production (Step 2) require different driving voltages (or power inputs), which implies that we can flexibly use renewable energy, such as solar or wind power, to produce H_2_ or O_2_ based on the output variation in these unstable power sources. For example, we can use solar energy at noon to drive the H_2_-production step, which requires a high driving voltage (or power input), and solar energy at dusk to power the O_2_-production step, which requires a low driving voltage. The flexibility can increase the use of sustainable energy.

In the above investigation (Fig. 2), a step time of only 10 min (600 s) was used to characterize the separate H_2_ and O_2_ production. Such a short time was used to emphasize that we can flexibly change the operation of our system even within a notably short time. In fact, the electrolysis time in each step can be easily controlled by the applied current. As shown in Supplementary Fig. 10, the electrolysis time in each step can be increased to 12 h with a low current of 20 mA. In addition, the Ni(OH)_2_ electrode can be cycled with different charge depths (Supplementary Fig. 11). Thus, we can also control the electrolysis time in each step by adjusting the charge depths of the Ni(OH)_2_ electrode (Supplementary Fig. 12). However, as mentioned in the introduction section, alkaline water electrolysis can use non-precious electrodes for the H_2_/O_2_ production^36^,^37^. Therefore, non-precious electrodes (a Co_3_O_4_-based OER electrode and a metal-Ni-foam-based HER electrode) were used to further demonstrate the separate H_2_ and O_2_ production (see Supplementary Fig. 13 and Supplementary Movies 3 and 4 for details). Furthermore, according to the previous report by Cronin *et al*.^39^, the efficiency of two-step water electrolysis can be evaluated by comparing its total driving voltage (Step 1+Step 2) to the driving voltage of the corresponding one-step water electrolysis. Therefore, the efficiency of the two-step alkaline water electrolysis using precious or non-precious HER/OER electrodes was calculated according to the method described by Cronin *et al*. (see Supplementary Fig. 14 for detail). As shown in Supplementary Fig. 14a,b, the efficiency of the two-step water electrolysis using precious electrodes (a RuO_2_/IrO_2_-coated Ti-mesh electrode for the OER and a Pt-coated Ti-mesh electrode for the HER) is 92% (=1.829/1.985) compared with its corresponding one-step water electrolysis. According to the data shown in Supplementary Fig. 14c,d, the efficiency of the two-step water electrolysis using non-precious electrodes (a Co_3_O_4_-based electrode for the OER and a metal Ni-foam electrode for the HER) is also ∼92% (=1.973/2.137) compared with its corresponding one-step water electrolysis. The achieved efficiency is slightly higher than that (79%) of the two-step PEM water electrolysis reported by Cronin's group^39^.

### Purity of the generated H_2_/O_2_

**Figure 3:**
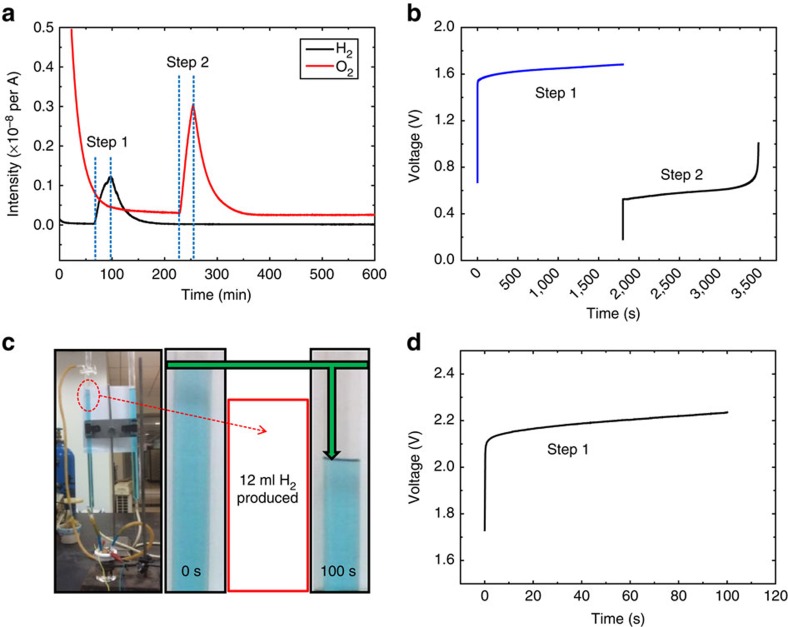
Purity of the generated H_2_/O_2_. (**a**) *In situ* differential electrochemical mass spectrometry (DEMS) curves of the H_2_ evolution (black line) and O_2_ evolution (red line) in the total water electrolysis process at a constant applied current of 200 mA. A pure Ar gas stream was used as a purge gas before electrolysis and the carrier gas in the total electrolysis process. There is a rest time of 130 min between Steps 1 and 2. (**b**) The chronopotentiometry curve (cell voltage versus time) of the electrolytic cell corresponds to the *in situ* DEMS test with two steps: H_2_-production process of 30 min (Step 1, the blue line) and O_2_-production process (Step 2, black line). (**c**) A typical apparatus configuration to determine the evolution of the H_2_ volumes. (The H_2_ production rate (ml s^−1^) was measured with an applied current of 1,000 mA for 100 s.) Approximately 12 ml H_2_ was generated after 100 s of electrolysis, as indicated by the green arrow. (**d**) The chronopotentiometry curve (cell voltage versus time) of the electrolytic cell corresponds to the H_2_-production volume test with an applied current of 1,000 mA for 100 s.

To confirm the purity of the H_2_/O_2_ in the separate steps, *in situ* differential electrochemical mass spectrometry was used to measure the gas evolution of the total water electrolysis process at a constant applied current of 200 mA. In this experiment, a quadrupole mass spectrometer with a leak inlet was connected to the alkaline water electrolytic cell with two tubes as the purge/carrier gas inlet and outlet (see the Methods and Supplementary Fig. 15 for details). A pure Ar gas stream was used as the purge gas before electrolysis and the carrier gas during the electrolysis process. Before the online gas analysis, the system was purged with a pure Ar stream for 1 h. The system was further purged with a pure Ar stream for another 1 h, with an online analysis record (Fig. 3a) showing that both O_2_ and H_2_ reached a stable background line. Then, the H_2_-production step (Step 1) was started, and the H_2_ evolution is clearly observed in the online analysis record. The ion current intensity of O_2_ obviously remained at the background level in Step 1, which indicates that no O_2_ was generated in the H_2_ production process of 30 min (Fig. 3a,b). After the H_2_ production (Step 1) finished, a rest step of 130 min was performed with a pure Ar stream to eliminate remnant H_2_ in the system, and a hysteresis of H_2_ could be observed in the online analysis record. Afterward, the O_2_-production step (Step 2) was started. As shown in Fig. 3b, the O_2_ production automatically finished with a total electrolysis time of ∼30 min (1,700 s), which is close to the H_2_ production time (1,800 s). The minor difference of 100 s may be because of the slight self-discharge of NiOOH in the rest step. However, this description does not indicate that the self-discharge of the NiOOH electrode will be significantly aggravated with a longer rest time (see Supplementary Fig. 16 for an extended discussion about the self-discharge of the nickel hydroxide electrode). As shown in Fig. 3a, there is no H_2_ evolution in the O_2_ production process. Therefore, the results in Fig. 3a,b well demonstrate the purity of the H_2_/O_2_ in the separate steps. Herein, it should be noted that the online gas analysis in our experiment was only used to characterize the purity of the H_2_ and O_2_ in separate steps. A typical drainage method (Supplementary Fig. 17) was used to quantify the H_2_ generation over a specific time length. In this experiment, the H_2_ production rate (ml s^−1^) was measured with an applied current of 1,000 mA for 100 s (Fig. 3c,d). Figure 3c,d shows that ∼12 ml H_2_ was generated in the 100 s electrolysis, which is close to the theoretical value (12.67 ml). Therefore, the Faradaic efficiency is 94.7% (12/12.67). In theory, the Faradaic efficiency should be 100%, but the impedance and dissolution of H_2_ in the aqueous solution may slightly reduce the efficiency. This method was used to measure the generated O_2_ volume in Step 2 at the identical current of 1,000 mA. The obtained result indicates that ∼6 ml O_2_ was generated in Step 2. Therefore, the H_2_-to-O_2_ ratio is 2:1 in the consecutive cycle of Steps 1 and 2.

### Combination between the H_2_-production and NiOOH-Zn battery

**Figure 4:**
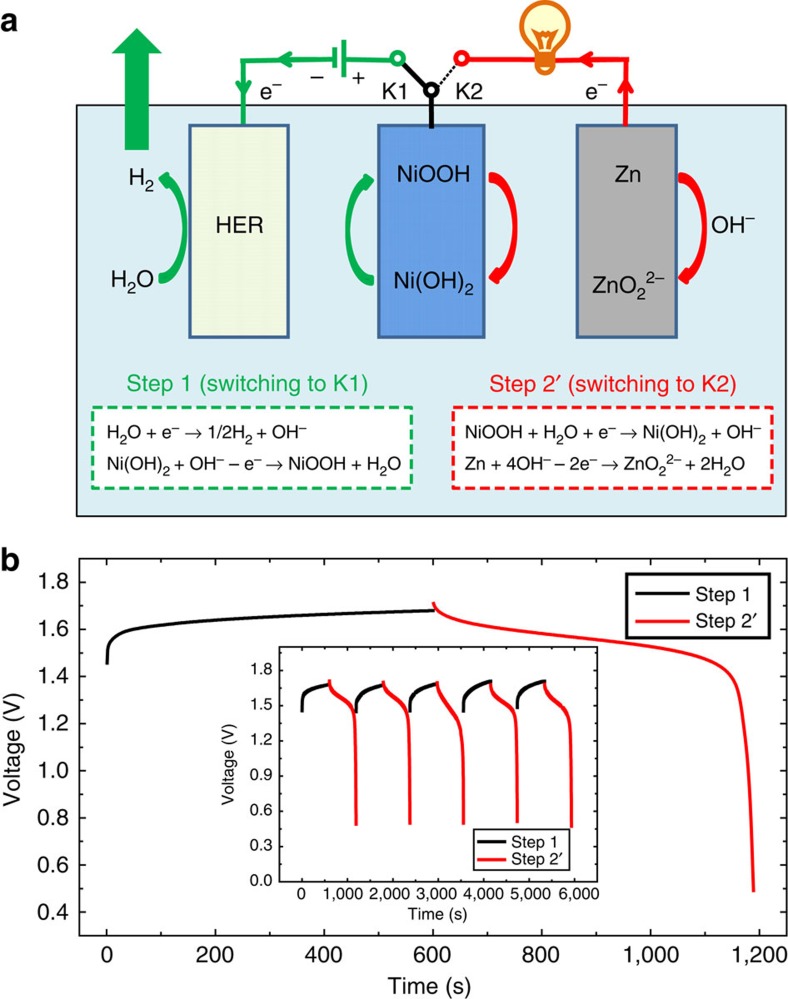
Combination of the H2-production and the NiOOH-Zn battery. (**a**) A schematic of the operation mechanism of the cell. (Herein, the H_2_-production process (Step 1; switching to K1) includes the cathodic reduction of H_2_O on the HER electrode (H_2_O+e^−^→1/2H_2_+OH^−^) and the anodic oxidization of the Ni(OH)_2_ electrode (Ni(OH)_2_+OH^−^— e^−^→ NiOOH+H_2_O). Then, by switching to K2, the NiOOH electrode that was formed during Step 1 is coupled with a zinc anode to form a NiOOH-Zn battery, and its discharge (Step 2′) depends on the cathodic reduction of the NiOOH electrode (NiOOH+H_2_O+e^−^→Ni(OH)_2_+OH^−^) and the anodic oxidization of the zinc electrode (Zn+4OH^−^—2e^−^→ZnO_2_^2−^+2H_2_O)). (**b**) Chronopotentiometry curve (cell voltage versus time) of the H_2_-production process (Step 1, black line) and the discharge curve of the NiOOH-Zn battery (Step 2′, red line). The electrolysis for H_2_ production applied a current of 200 mA for 600 s; then, the discharge profile of the NiOOH-Zn battery was also investigated with a current of 200 mA. The inset is the cycle performance of the H_2_-production step (black line) and discharge step (red line) of the NiOOH-Zn battery with an applied current of 200 mA. As shown in **a**,**b**, Step 1 requires a power input to produce H_2_ gas, whereas Step 2′ (discharge of the NiOOH-Zn battery) can deliver energy to power other devices.

Interestingly, the aforementioned O_2_-production step (Step 2) can be replaced by the discharge step of the NiOOH-Zn battery (Step 2′), which will enable the coupling of H_2_ production with a discharge step of the NiOOH-Zn battery (Fig. 4a). As shown in Fig. 4a, the H_2_ production (Step 1) includes the cathodic reduction of H_2_O on the HER electrode (H_2_O→H_2_) and the anodic oxidization of the Ni(OH)_2_ electrode (Ni(OH)_2_→NiOOH). Next, the NiOOH electrode that is formed in Step 1 is coupled with a zinc anode to form a NiOOH-Zn battery. The subsequent discharge step (Step 2′) of the NiOOH-Zn battery is based on the cathodic reduction of the NiOOH electrode (NiOOH→Ni(OH)_2_) and the anodic oxidization (Zn→ZnO_2_^2−^) of zinc^48^,^49^,^50^. In other words, the architecture in Fig. 4a provides an interesting rechargeable cycle that produces H_2_ with charge (that is, electrolysis in Step 1) and delivers energy with the discharge of the NiOOH-Zn battery (Step 2′). To confirm this hypothesis, the NiOOH electrode, which formed after the electrolysis for H_2_ production with an applied current of 200 mA for 600 s, was directly coupled with a zinc-plate electrode in the electrolysis cell to construct a NiOOH-Zn battery. The discharge profile of the NiOOH-Zn battery was investigated with a current of 200 mA. As shown in Fig. 4b, the NiOOH-Zn battery displays a discharge voltage of ∼1.6 V with a total discharge time of ∼600 s. Furthermore, the consecutive H_2_-production step (Step 1) and discharge step (Step 2′) of the NiOOH-Zn battery can be cycled exactly like a rechargeable battery (inset of Fig. 4b). Further cycle data of Step 1 (H_2_-production step) and Step 2′ (discharge of the NiOOH-Zn battery) are provided in Supplementary Fig. 18. Therefore, the architecture in Fig. 4a introduces a new energy storage/conversion approach, in which solar energy can be used to drive the electrolysis (charge process) of Step 1 to produce H_2_ during the daytime, and the discharge step (Step 2′) of the NiOOH-Zn battery can be used to deliver energy to power electronic devices overnight. To clarify this point for the layperson reader, the NiOOH-Zn battery that formed after the H_2_-production step was used to power a 1.6 V electric fan (Supplementary Movie 5). The cycle of Steps 1 and 2′ also increases the Zn(OH)_2_ concentration in the alkaline electrolyte. The Zn(OH)_2_ alkaline solution can be used to produce O_2_ and metallic Zn through electrolysis at the proper time with other energy inputs, such as at night-time with wind power, nuclear fission and so on. In addition, the rechargeable system based on the H_2_ production (Step 1) and discharge of the NiOOH-Zn battery (Step 2′) exhibits a theoretical energy density of 280 Wh kg^−1^ (see Supplementary Fig. 19 for details), which is close to the theoretical energy density of conventional Ni-MH batteries, Ni-Cd batteries or Ni-Zn batteries and higher than the theoretical energy density of lead-acid batteries and aqueous Li-ion batteries^51^.

## Discussion

We successfully split the conventional alkaline electrolysis into two steps by using nickel hydroxide as a recyclable redox mediator. The separate H_2_ and O_2_ production overcomes the challenge of H_2_/O_2_ mixing and facilitates the operation of alkaline electrolysis even with unstable power inputs. The separate H_2_ and O_2_ productions require different driving voltages (or input power), which implies that we can flexibly use sustainable energy, such as solar or wind power, with higher efficiency. Finally, the combination of H_2_ production and discharge of the NiOOH-Zn battery potentially provides a new energy storage/conversion approach for human life. It should also be noted that the electrochemical redox process of Ni(OH)_2_/NiOOH is generally limited by the proton diffusion in the crystalline framework of Ni(OH)_2_ or NiOOH^52^, which limits the electrolysis rate. Therefore, solar energy, with the characteristic of low power loads, should be suitable to drive the new type alkaline electrolytic cell. However, much effort has been made to develop high-rate Ni(OH)_2_ electrodes to increase the power density of Ni(OH)_2_-based batteries, which may further enhance the electrolysis rate of the new type alkaline electrolytic cell. The data in the result section were obtained using a one-compartment water electrolytic cell, where all three electrodes (HER cathode, OER anode and Ni(OH)_2_ electrode) were immersed in the same electrolyte. In practical applications, the two-step alkaline water electrolysis can also be operated with two separate rooms, where the nickel hydroxide (Ni(OH)_2_/NiOOH) electrode is used as ‘a solid-state proton buffer' (see the proton buffer mechanism in Supplementary Fig. 1), which can be moved between room 1 for H_2_ production (Step 1) and room 2 for O_2_ production (Step 2; see the extended discussion in Supplementary Figs 20 and 21 and Supplementary Movies 6 and 7).

## Methods

### Synthesis and characterization of Ni(OH)_2_/MWNT composite

First, multi-walled carbon nanotubes (MWNTs) underwent a hydrophilic treatment before use. The MWNTs were sonicated in 30% HNO_3_ solution for 30 min, filtered and washed with distilled water, and finally dried at 100°C for 12 h. According to our previous report, Ni(OH)_2_/MWNT composites were prepared by loading Ni(OH)_2_ on the treated MWNTs in an alkaline medium: 0.3 g of accurately weighed MWNTs was immersed and dispersed in a bath that contained 2.2 g of 0.1 M Ni(NO_3_)_2_·6H_2_O solution. Then, 0.1 M KOH solution was dropped into the bath while stirring until the pH of the aqueous solution was 8.5. The resulted product was filtered and repeatedly washed with distilled water, dried at 80 °C and weighed. The 70 wt % mass load of Ni(OH)_2_ in the composites was evaluated by calculating the weight difference of MWNTs. The Ni(OH)_2_/MWNT composite was characterized using S-4800 scanning electron microscopes and a Joel JEM2011 transmission electron microscope.

### Preparation and electrochemical test of the electrode

The Ni(OH)_2_/MWNT composite electrode was prepared according to the following steps: a mixture of 85 wt% Ni(OH)_2_/MWNT composites, 10 wt% acetylene black and 5 wt% polytetrafluoroethylene was thoroughly mixed to form a film, which was pressed onto a nickel grid (1.2 × 10^7^ Pa) that served as a current collector surface (1 cm^2^). The Ni(OH)_2_/MWNT composite electrode was characterized by CV with a scan rate of 5 mV s^−1^ and a galvanostatic charge-discharge test at a current density of 0.2 A g^−1^. The electrolyte was 1 M KOH solution. The onset potential of the OER on the commercial RuO_2_/IrO_2_-coated Ti-mesh electrode and onset potential of the HER on the commercial Pt-coated Ti-mesh electrode in 1 M KOH were investigated by linear sweep voltammetric measurements with a sweep rate of 5 mV s^−1^ in 1 M KOH. The aforementioned experiments were performed with a typical three-electrode method, in which a Pt plate and Hg/HgO (0.098 V versus the standard hydrogen electrode) were used as the counter and reference electrodes, respectively. All electrochemical measurements were performed with a CHI 660D electrochemistry workstation.

### Fabrication of the new type electrolytic cell

The cell was constructed with a commercial Pt-coated Ti-mesh electrode (Supplementary Fig. 4) for the HER, a commercial RuO_2_/IrO_2_-coated Ti-mesh electrode for the OER (Supplementary Fig. 5) and a commercial Ni(OH)_2_ electrode of conventional Ni-MH or Ni-Cd batteries (Supplementary Fig. 6). The photo profile of the cell is shown in Supplementary Fig. 7, where the Ni(OH)_2_ electrode (2.5 × 4 cm^2^) is located between the HER electrode (2.5 × 4 cm^2^) and OER electrode (2.5 × 4 cm^2^).

### Water electrolysis investigation

The water electrolysis of the new type alkaline water electrolysis cell was investigated using chronopotentiometry measurements with applied currents of 100, 200 and 500 mA. In Step 1, the HER electrode (that is, Pt coated Ti-mesh electrode) and Ni(OH)_2_ electrode were connected to the cathode and anode, respectively, of the power source for electrolysis. The duration time of Step 1 (that is, the H_2_-production step) is 600 s with an applied current of 100, 200 or 500 mA. After Step 1, Step 2 (that is, the O_2_-production step) was started. In Step 2, the NiOOH electrode, which formed during Step 1, and the OER electrode (that is, the RuO_2_/IrO_2_-coated Ti-mesh electrode) were connected to the cathode and anode, respectively, of the power source for the electrolysis. Step 2 automatically stopped when the cell voltage sharply increased at the end of the electrolysis, which indicated that all the NiOOH was converted into Ni(OH)_2_. The cell voltages (*V* versus time) of Steps 1 and 2 were recorded to characterize the electrolysis profile. With an additional reference electrode (that is, the Hg/HgO electrode), the chronopotentiometry data (potential versus time) of a single electrode (the HER electrode, Ni(OH)_2_ electrode or OER electrode) were also recorded during Steps 1 and 2. A schematic illustration of the voltage (or potential) record is provided in Supplementary Fig. 22. As shown in Supplementary Fig. 22, *V*_1_ is the cell voltage in Step 1; *V*_2_ is the cell voltage in Step 2; *P*_1_ is the potential of the HER electrode in Step 1; *P*_2_ is the potential of the Ni(OH)_2_ electrode in Steps 1 and 2 and *P*_3_ is the potential of the OER in Step 2. A PARSTAT MC multi-channel workstation was used to perform the water electrolysis investigation of Fig. 2.

### *In situ* differential electrochemical mass spectrometry

A quadrupole mass spectrometer (NETZSCH QMS 403 C) with a leak inlet was applied to measure the gas evolution of the total water electrolysis process at a constant applied current of 200 mA. The electrolysis process was performed using a Solarton Instrument Model 1287 electrochemical interface. As shown in Supplementary Fig. 15, the mass spectrometer is connected to the new-type alkaline water electrolysis cell with two tubes as the purge/carrier gas inlet and outlet. A pure Ar gas stream was used as the purge gas before electrolysis and the carrier gas during the electrolysis process. The gas flows were typically 10 ml min^−1^. Before the online gas analysis, the system was purged with a pure Ar stream for 1 h. The system was further purged with a pure Ar stream for another 1 h before Step 1 began. The duration time of Step 1 was 30 min. After the end of Step 1, a rest step of 130 min was performed with a pure Ar stream to eliminate remnant H_2_ in the system. Then, Step 2 was started and continued until the cell voltage sharply increased. The experiment completed when all remaining O_2_ in the electrolysis cell was removed.

### Data availability

All relevant data are available from the authors.

## Additional information

**How to cite this article:** Chen, L. *et al*. Separating hydrogen and oxygen evolution in alkaline water electrolysis using nickel hydroxide. *Nat. Commun.* 7:11741 doi: 10.1038/ncomms11741 (2016).

## Supplementary Material

Supplementary Information Supplementary Figures 1-22 and Supplementary References

Supplementary Movie 1 Supplementary Movie 1 shows the H_2_ production process (Step1) using precious (Pt-coated Ti-mesh) HER electrode. The applied current is 300mA, and the electrolysis duration time is 300 seconds. In the Supplementary Movie 1, it is obviously that the bubbles continuously evolved and released from the surface of HER electrodes when the electrolysis began, and throughout the whole H_2_ generation process, no O_2_ production was observed.

Supplementary Movie 2 Supplementary Movie 2 shows the O_2_ production process (Step2) using precious (RuO_2_/IrO_2_-coated Ti-mesh) OER electrode. The applied current is 300mA, and the duration time is about 300 seconds. No Hydrogen evolution reaction was detected during the O_2_ production process according to Supplementary Movie 2.

Supplementary Movie 3 Supplementary Movie 3 shows the H_2_ production process (Step1) using the non-precious (Ni-foam) HER electrode. The applied current is 300mA, and the electrolysis duration time is 300 seconds. It is obvious that the bubbles continuously evolved and released from the surface of HER electrodes when the electrolysis began, and throughout the whole H_2_ generation process, no O_2_ production was observed.

Supplementary Movie 4 Supplementary Movie 4 shows the O_2_ production process (Step2) using the non-precious (Co_3_O_4_) OER electrode. The applied current is 300mA, and the duration time is about 300 seconds. No Hydrogen evolution reaction was detected during the O_2_ production process according to Supplementary Movie 4.

Supplementary Movie 5 Supplementary Movie 5 shows the NiOOH-Zn battery formed after H_2_-production step, and it was used to power a 1.6 V electric fan.

Supplementary Movie 6 Supplementary Movie 6 shows the H_2_ production process (Step1) in room-1 using precious (Pt coated on Ti-mesh) electrode with a commercial Ni(OH)_2_ electrode. The applied current is 300mA, and the electrolysis duration time is 300 seconds.

Supplementary Movie 7 Supplementary Movie 7 shows the O_2_ production process (Step2) in room-2 using precious (RuO_2_/IrO_2_ coated on Ti-mesh) electrode and the NiOOH electrode from room-1 after H_2_ production process. The applied current is 300mA, and the electrolysis duration time is 300 seconds.

Peer review file
