# Supplementary material for: Separating hydrogen and oxygen evolution in alkaline water electrolysis using nickel hydroxide
Source: Nat Commun. 2016 May 20;7:11741. doi: 10.1038/ncomms11741 (PMC4876480; doi:10.1038/ncomms11741)
Supplement: Supplementary Information — Supplementary Figures 1-22 and Supplementary References [file ncomms11741-s1.pdf]

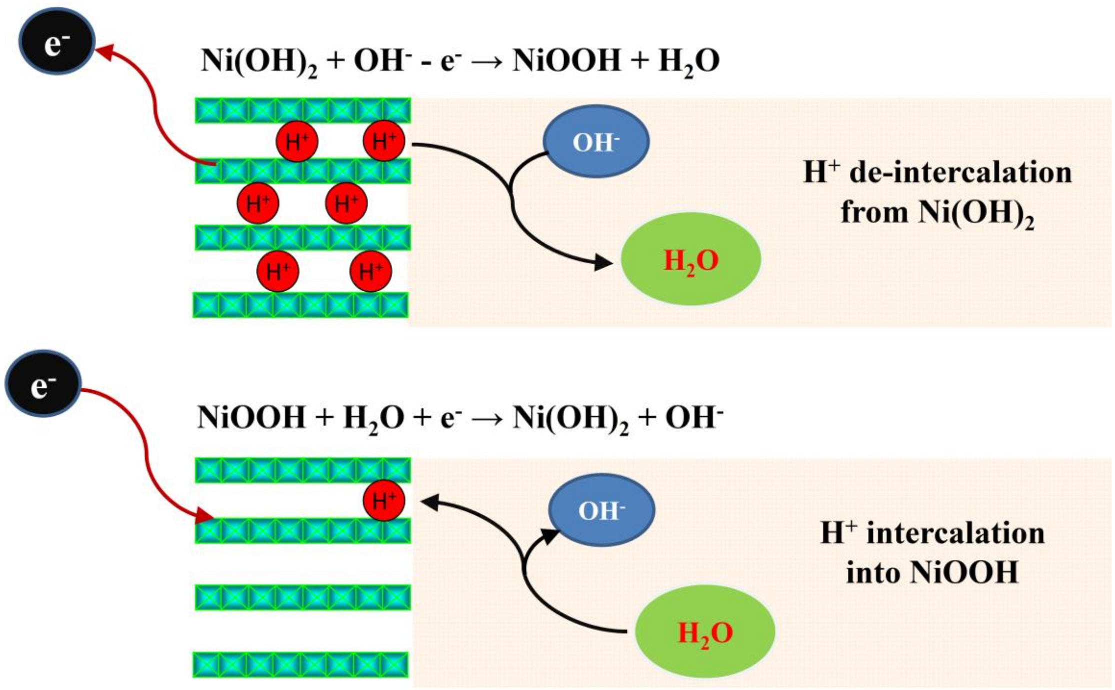

Supplementary Figure 1 | Schematic illustration of proton release and storage during the charge/discharge process of nickel hydroxide. As shown in supplementary Figure 1, the reversible transformation of $Ni(OH)_2/NiOOH$ can be explained as the electron-coupled proton release and storage process (i.e. H^+ de-intercalation from $Ni(OH)_2$ and H^+ intercalation into NiOOH).

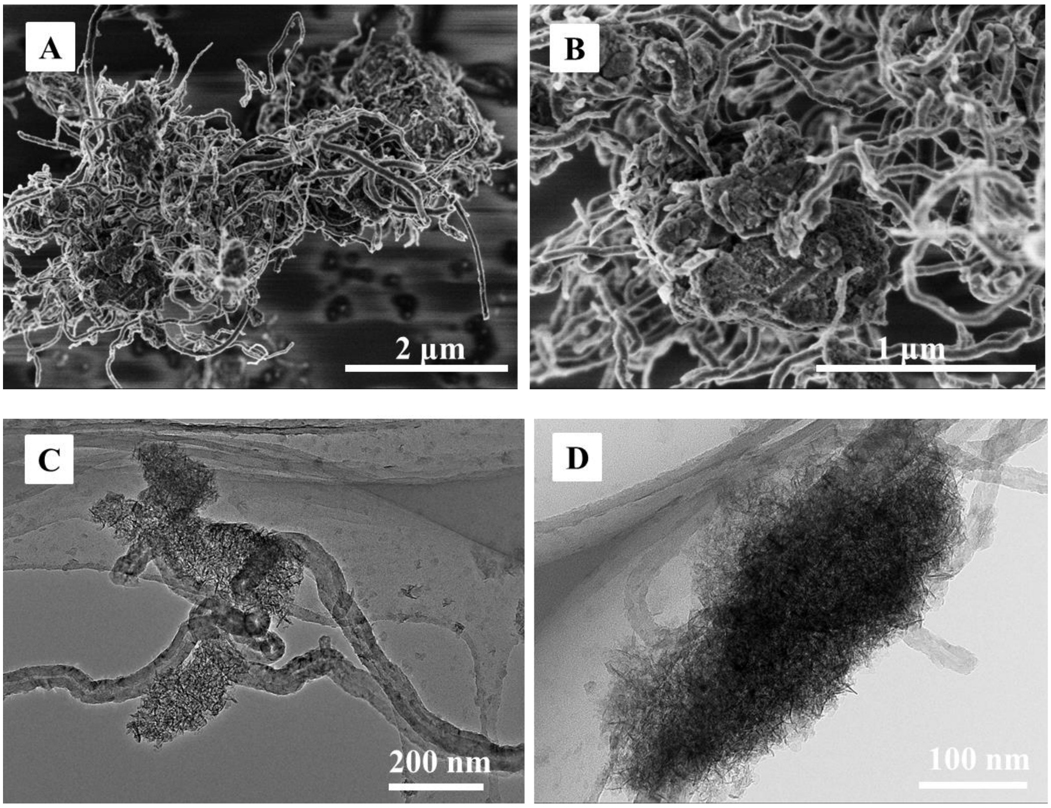

Supplementary Figure 2 | SEM and TEM images of $Ni(OH)_2$/MWNT composites. Supplementary Figure 2A and 2B give the SEM images of $Ni(OH)_2$/MWNT composites. The SEM images clearly show that the $Ni(OH)_2$ is well distributed on the netlike structure MWNTs. Supplementary Figure 2C and 2D show the TEM images of the $Ni(OH)_2$/MWNTs composites. Herein, the CNTs supporter with high electronic conductivity is just used to alleviate the polarization arising from electrode impedance in CV investigation.

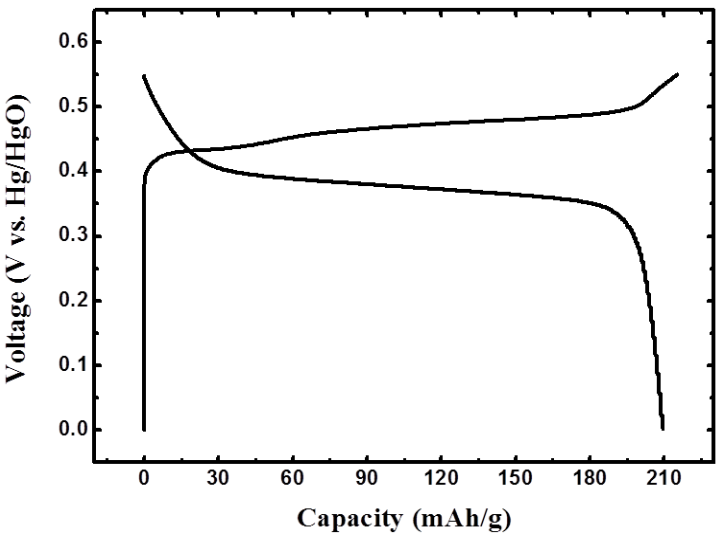

Supplementary Figure 3 | Galvanostatic charge–discharge curve of Ni(OH)$_2$/MWNT. Supplementary Figure 3 shows the typical galvanostatic charge–discharge curve of electrode based on Ni(OH)$_2$/MWNT composites at a current density of 0.2 A/g within a potential window of 0-0.55V (vs. Hg/HgO). As shown in Supplementary Figure 3, a flat plateau at about 0.45V (vs. Hg/HgO) and 0.38V (vs. Hg/HgO) was observed in the charge and discharge profile respectively, corresponding to the Ni(OH)$_2$ oxidization (Ni(OH)$_2$ + OH$^-$ - e$^-$→ NiOOH + H$_2$O) and the NiOOH reduction (NiOOH + H$_2$O + e$^-$ → Ni(OH)$_2$ + OH$^-$). The specific capacity is about 210mAh/g, which is calculated according to the total weight of both Ni(OH)$_2$ and MWNTs.

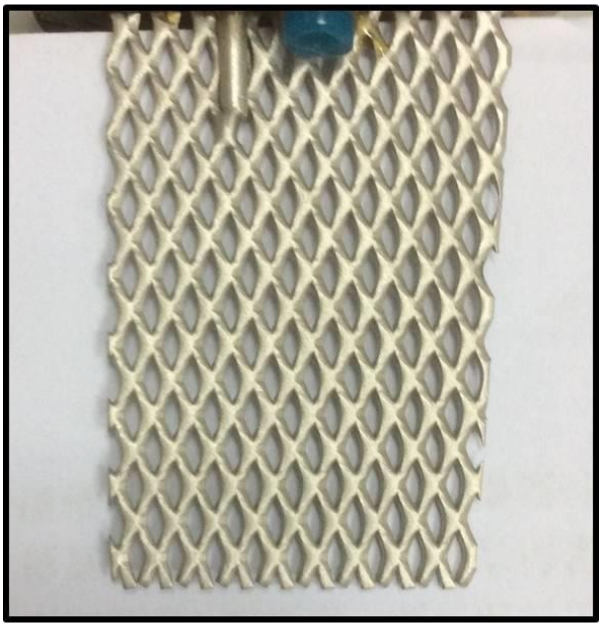

Supplementary Figure 4 | Photograph of the commercial Pt coated Ti-mesh electrode. The size of this mesh electrode is 2.5×4 cm^2, and it is purchased from Baoji Zhiming Special Metal Co., LTD (China). [Tel.86-0917-3122785; http://www.zmanode.com]

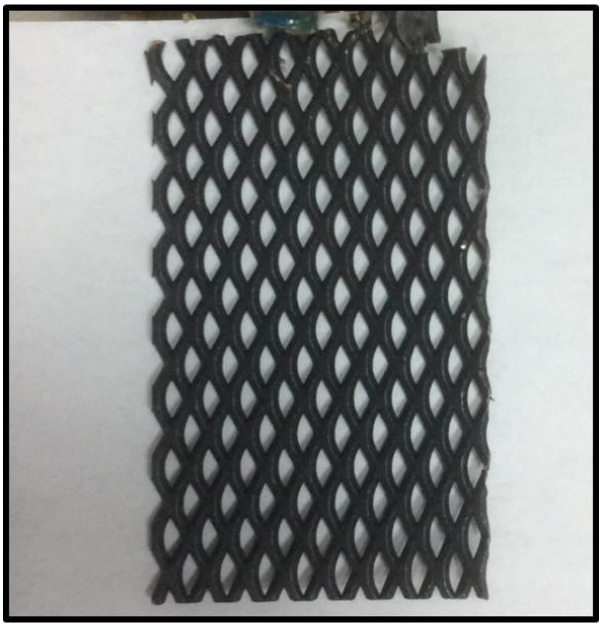

Supplementary Figure 5 | Photograph of the commercial RuO_2/IrO_2 coated Ti-mesh electrode. The size of this mesh electrode is 2.5×4 cm^2, and it is purchased from Baoji Zhiming Special Metal Co., LTD [Tel.86-0917-3122785; http://www.zmanode.com].

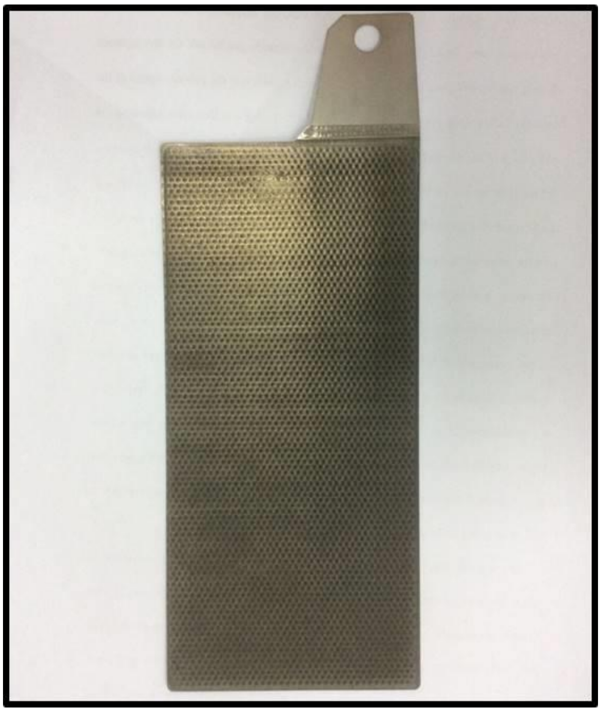

Supplementary Figure 6 | Photograph of the commercial $Ni(OH)_2$ electrode. Supplementary Figure 6 gives the photograph of the commercial $Ni(OH)_2$ electrode of conventional Ni-MH or Ni-Cd batteries [obtained from Shanghai Aowei Technology Development Co., Ltd; http://www.aowei.com]. The pristine size of the electrode is 18×8 cm^2. It was cut into the size of 2.5×4 cm^2 for fabricating the new type alkaline electrolytic cell.

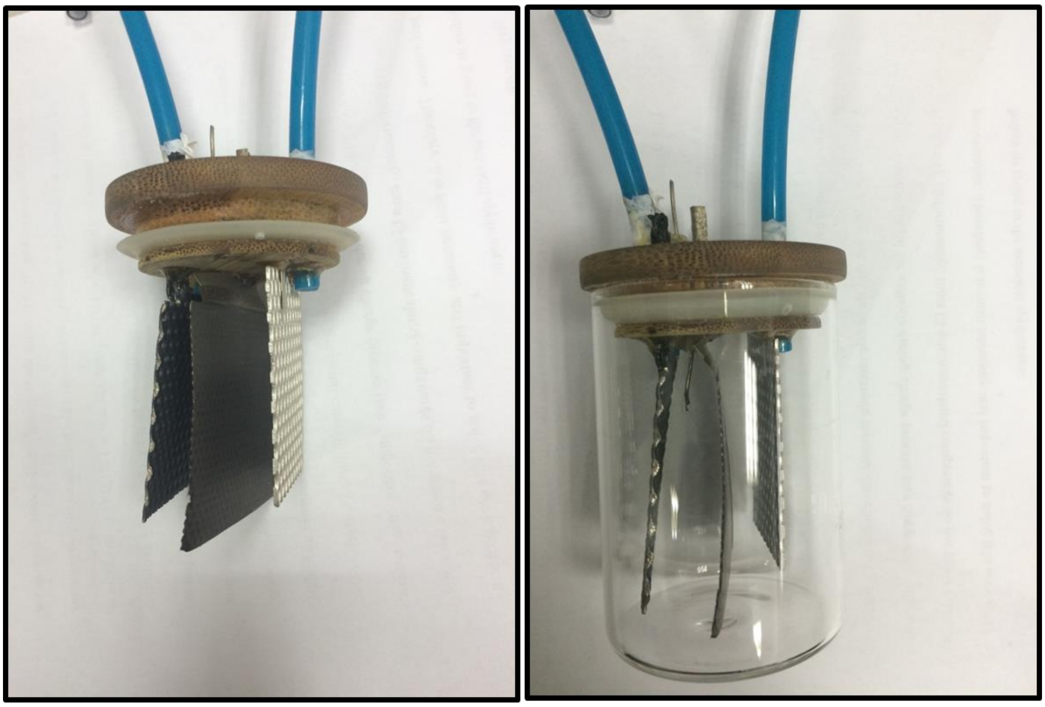

Supplementary Figure 7 | Photo profile of the new type alkaline water electrolysis cell. It was constructed with a commercial Pt coated Ti-mesh electrode (Supplementary Figure 4) for HER, a commercial RuO_2/IrO_2 coated Ti-mesh electrode for OER (Supplementary Figure 5) and a commercial $Ni(OH)_2$ electrode of conventional Ni-MH or Ni-Cd batteries (Supplementary Figure 6). It can be detected that the $Ni(OH)_2$ electrode is located between the HER electrode and OER electrode. In addition, there are two gas channel tubes as gas inlet and outlet in this electrolysis cell.

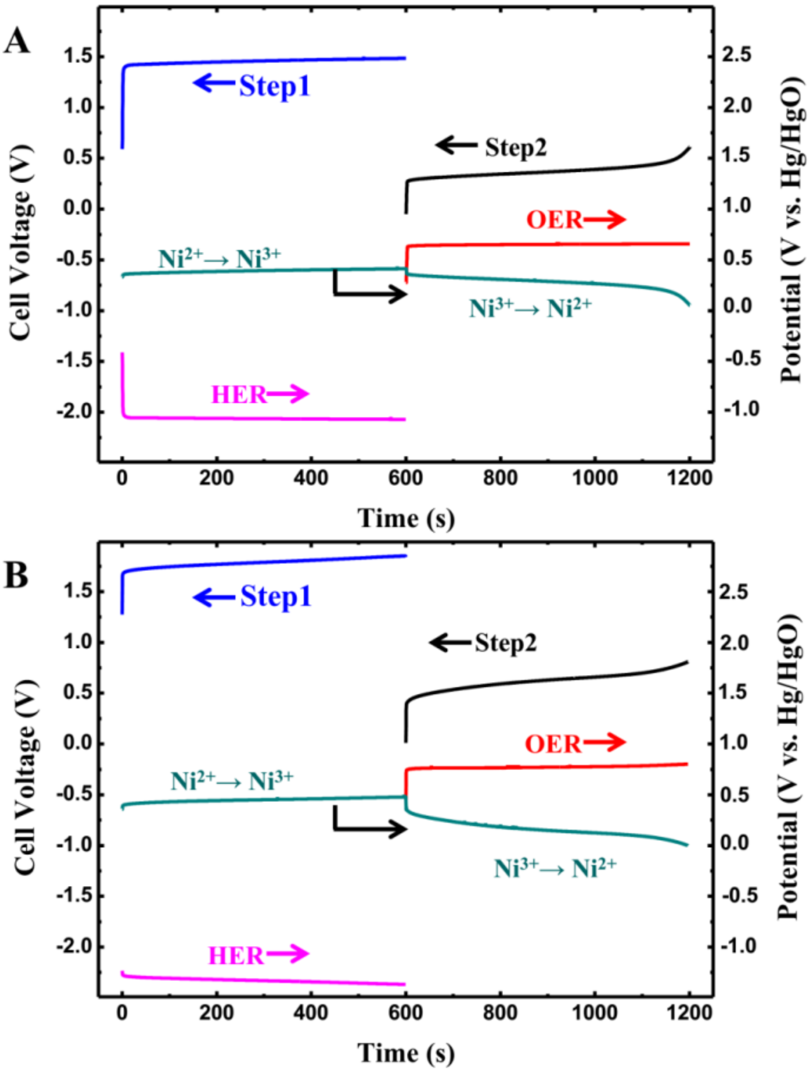

Supplementary Figure 8 | Chronopotentiometry curve (cell voltage vs. time) of the electrolysis cell at different applied currents. Supplementary Figure 8 gives the Chronopotentiometry curve (cell voltage vs. time) of the electrolysis cell at a constant applied current of: (**A**) 100 mA (**B**) 500mA in 1M KOH with two steps: Step 1 is H_2 production process (the blue line); Step 2 is O_2 production process (the black line). Supplementary Figure 8 also shows the Chronopotentiometry curve of the HER electrode (potential vs. time, the pink line), Chronopotentiometry curve of the $Ni(OH)_2$ electrode (potential vs. time, the green line) and the Chronopotentiometry curve of the OER electrode (potential vs. time, the red line). As shown in Supplementary Figure 8A, step 1 (i.e. H_2 production process) exhibits a cell voltage of about 1.5 V, which arises from the difference between the anodic potential 0.4V (vs. Hg/HgO) of $Ni(OH)_2$ oxidation ($Ni(OH)_2 \rightarrow$ NiOOH) and the cathodic potential -1.1V (vs. Hg/HgO) of H_2O reduction ($H_2O \rightarrow H_2$). In consequent step 2 (i.e. O_2 production process), the cell voltage is 0.3 V, which is equal to the potential difference (0.6- 0.3 V vs. Hg/HgO) between anodic oxidation of OH^- ($OH^- \rightarrow O_2$) and the cathodic reduction of NiOOH (NiOOH $\rightarrow Ni(OH)_2$). While when it was investigated at the current of 500 mA (Supplementary Figure 8B), the step 1 exhibits a higher cell voltage of

about 1.8V, which arises from the higher anodic potential 0.5V of $Ni(OH)_2$ oxidation and the lower cathodic potential -1.3V of H_2O reduction. In step 2, the cell voltage is 0.65V. the higher cell voltage is because of the bigger difference (0.75- 0.1 V) between anodic oxidation of OH^- and the cathodic reduction of NiOOH.

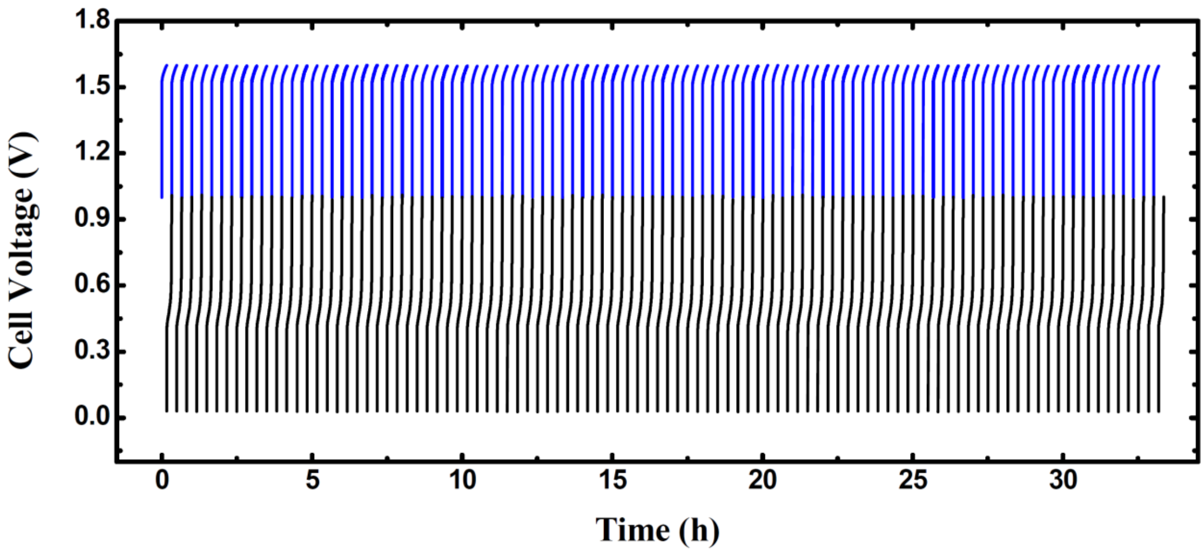

Supplementary Figure 9 | Electrochemical profile of 100 cycles of H_2/O_2 operation [Test current: 200 mA; step-time: 600 s; cell structure: Pt coated Ti-mesh electrode (2.5×4 cm^2) for HER / $Ni(OH)_2$ electrode (2.5×4 cm^2) / RuO_2/IrO_2 coated Ti-mesh electrode (2.5×4 cm^2) for OER].]

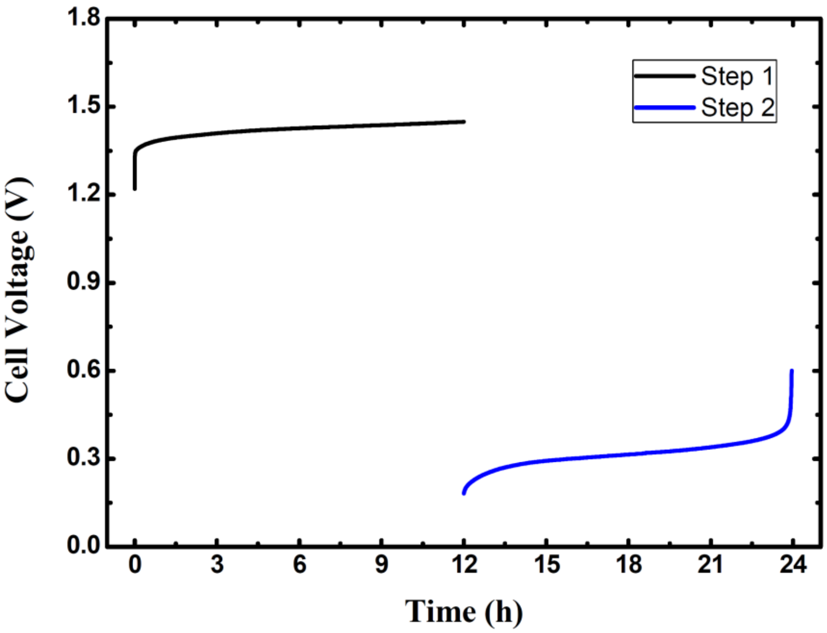

Supplementary Figure 10 | Electrochemical profile of the new type alkaline water electrolytic cell with a step-time of 12 hours and an applied current of 20 mA.[Cell structure: Pt coated Ti-mesh electrode (2.5×4 cm^2) for HER / $Ni(OH)_2$ electrode (2.5×4 cm^2) / RuO_2/IrO_2 coated Ti-mesh electrode (2.5×4 cm^2) for OER] As shown in Supplementary Figure 10**,** the electrolytic cell can be cycled with a step-time of 12 hours, which is corresponding to the daytime and nighttime. Higher operating current with such step-time (12 hours) can also be easily achieved by increasing the amount of $Ni(OH)_2$ electrode.

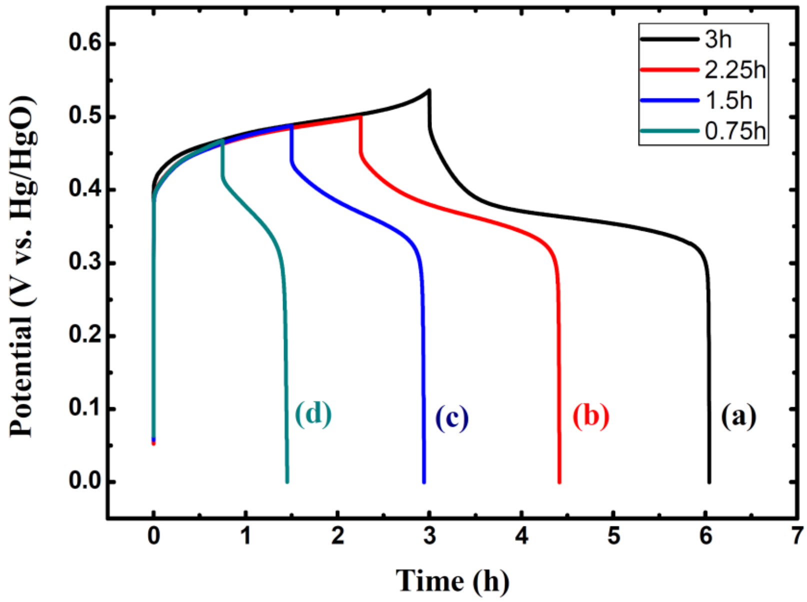

Supplementary Figure 11 | Electrochemical profile of a $Ni(OH)_2$ electrode (2.5×4 cm^2) at different charge depths tested with three-electrode method at a charge/discharge current of 100 mA. [Work electrode: $Ni(OH)_2$ electrode; Counter electrode: Pt coated Ti-mesh (2.5×4 cm^2); Reference electrode: Hg/HgO; Electrolyte: 1 M KOH].

In this experiment, electrochemical profile of a commercialized $Ni(OH)_2$ electrode (2.5×4 cm^2) was investigated with different charge depths through a typical three-electrode system [Work electrode: $Ni(OH)_2$ electrode; Counter electrode: Pt coated Ti-mesh (2.5×4 cm^2); Reference electrode: Hg/HgO; Electrolyte: 1 M KOH]. As shown in Supplementary Figure 11a, the $Ni(OH)_2$ electrode was charged with an applied current of 100 mA for 3 hours to reach the full charge depth, and then the electrode was discharged to 0 V (vs. Hg/HgO) with a discharge current of 100 mA. It can be detected that the corresponding discharge time (Supplementary Figure 11a) is 3 hours (= charge time), indicating a reversible cycle. When the charge depths are controlled at 75% (2.25 hours; Supplementary Figure 11b), 50% (1.5 hours; Supplementary Figure 11c) and 25% (0.75 hour; Supplementary Figure 11d), highly reversible charge/discharge profiles still can be observed, clearly. The results shown in Supplementary Figure 11 demonstrate that $Ni(OH)_2$ electrode can work well at various charge depths.

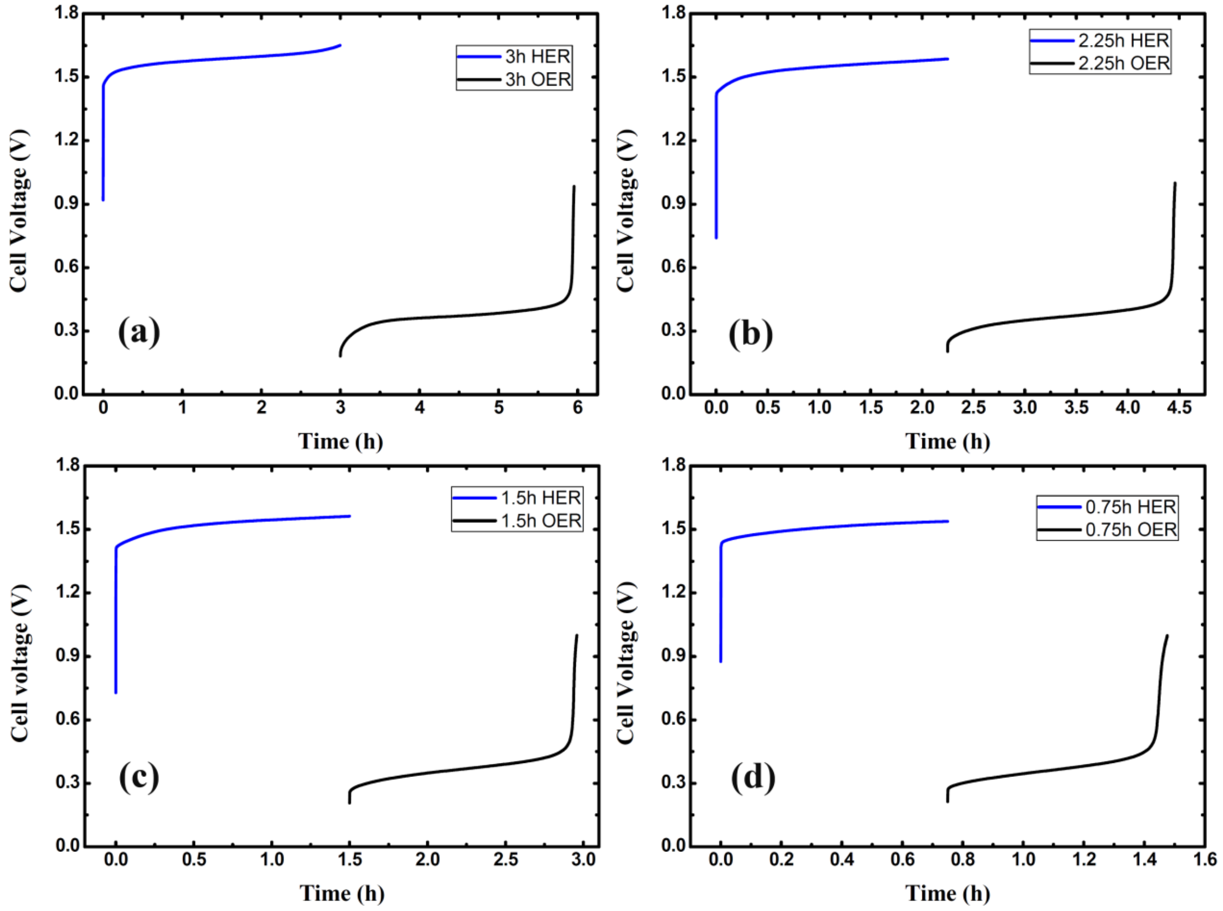

Supplementary Figure 12 | Electrochemical profile of the new type alkaline water electrolytic cell with different step-time and an applied current of 100 mA. (a) 3 hours, (b) 2.25 hours, (c) 1.5 hours and (d) 0.75 hour. [Cell structure: Pt coated Ti-mesh electrode (2.5×4 cm^2) for HER/ $Ni(OH)_2$ electrode (2.5×4 cm^2) / RuO_2/IrO_2 coated Ti-mesh electrode (2.5×4 cm^2) for OER].

According to the result shown in Supplementary Figure 11, we can easily control the step-time for H_2 production and O_2 production in the new type alkaline electrolytic cell by adjusting the charge depths of $Ni(OH)_2$ electrode. To clarify this point, charge-time of 3 hours, 2.25 hours, 1.5 hours and 0.75 hours of $Ni(OH)_2$ electrode with an applied current of 100 mA were employed to control the step-time for H_2 production and O_2 production in alkaline electrolysis electrolytic cell (Supplementary Figure 12).It can be observed from Supplementary Figure 12 that the new type alkaline electrolytic cell still can work efficiently.

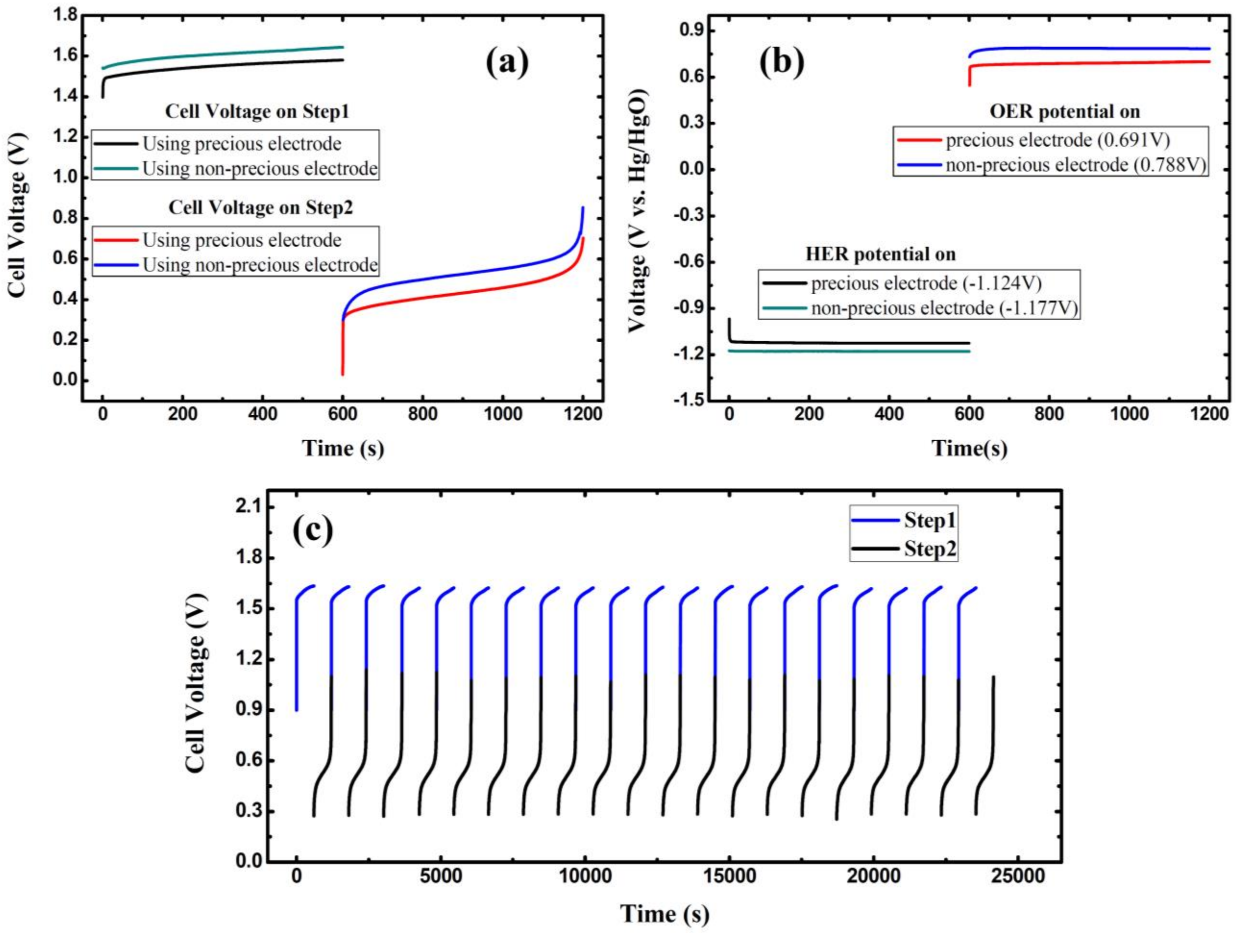

Supplementary Figure 13 | Electrochemical profile of separate H_2 and O_2 production in alkaline electrolytic cell using non-precious electrodes (Co_3O_4 for OER and Ni-foam for HER). (a) Cell voltages on step 1 and step 2 of the cells using non-precious electrodes and precious electrodes. (b) OER potentials on Co_3O_4-based electrode and RuO_2/IrO_2-based electrode and HER potentials on Ni-foam electrode and Pt-based electrode. (c) Chronopotentiometry curve of H_2 /O_2 generation cycle with an applied current 200 mA of the cell using non-precious electrodes.

According to the review article [1], Co_3O_4-based anode for OER and metal Ni-based cathode for HER have been widely applied for alkaline water electrolysis. Therefore, we employed Co_3O_4 and Ni-foam as OER and HER electrodes, respectively, to further investigate the separate steps. In this experiment, commercialized Co_3O_4 powder was treated by ball milling for 4 hours, and then was used to fabricate OER electrode. The Co_3O_4-based OER electrode was obtained by mixing 80 wt % Co_3O_4 powder, 10 wt % Ketjen Black (KB) as conductive agent, and 10 wt % polytetrafluoroethylene (PTFE) as binder. For a typical preparation, Co_3O_4, KB, and PTFE were dissolved in isopropanol to form a slurry with the weight ratio mentioned above, and then the slurry was rolled into a film. Finally, the film was pressed on stainless steel mesh to form OER electrode (2.5×4 cm^2). Commercialized Ni-foam was directly used as the HER electrode (2.5×4 cm^2). An alkaline water electrolytic cell was constructed with a Co_3O_4-electode for OER, a commercial Ni-foam electrode for HER and a $Ni(OH)_2$ electrode (See Supplementary Movie 3 and 4). Water electrolysis of the cell was investigated by chronopotentiometry measurements with an applied current of 200 mA. Chronopotentiometry curve (cell voltage vs. time) of the electrolytic cell is shown in Supplementary Figure 13a. The chronopotentiometry curve of the cell using precious electrodes tested at the same condition is also shown in Supplementary Figure 13a for comparison. In addition, OER potential on Co_3O_4

electrode and HER potential on Ni-foam electrode were investigated, in comparison with that on precious electrodes (see Supplementary Figure 13b). It can be observed from Supplementary Figure 13a that when using non-precious electrodes, the electrolysis process still includes two separate steps (Step 1 and 2) with different cell voltages. However, the cell voltages of the cell using non-precious electrodes (0.526V on step 1 and 1.611 V on step 2) are higher than that of the cell using precious electrodes (0.432V on step 1 and 1.553 V on step 2), which is owing to the lower catalytic ability of non-precious electrodes. As shown in Supplementary Figure 13b, the OER potential on Co_3O_4 electrode (0.788 V vs. Hg/HgO) is higher than that on commercialized RuO_2/IrO_2 coated Ti-mesh (0.691 V vs. Hg/HgO). The HER potential on Ni-foam electrode (- 1.177 V vs. Hg/HgO) is lower than that on commercialized Pt coated Ti-mesh (-1.124V vs. Hg/HgO). The cycle of step 1 (H_2 production) and step 2 (O_2 production) in the cell using non-precious electrodes was also investigated with an applied current of 200 mA (Supplementary Figure 13c). The achieved cycle performance is similar to that achieved by the cell using precious electrodes. In addition, the video evidence was also given in Supplementary Movie 3 and 4 to confirm the separate H_2/O_2 generation in the cell using non-precious electrodes.

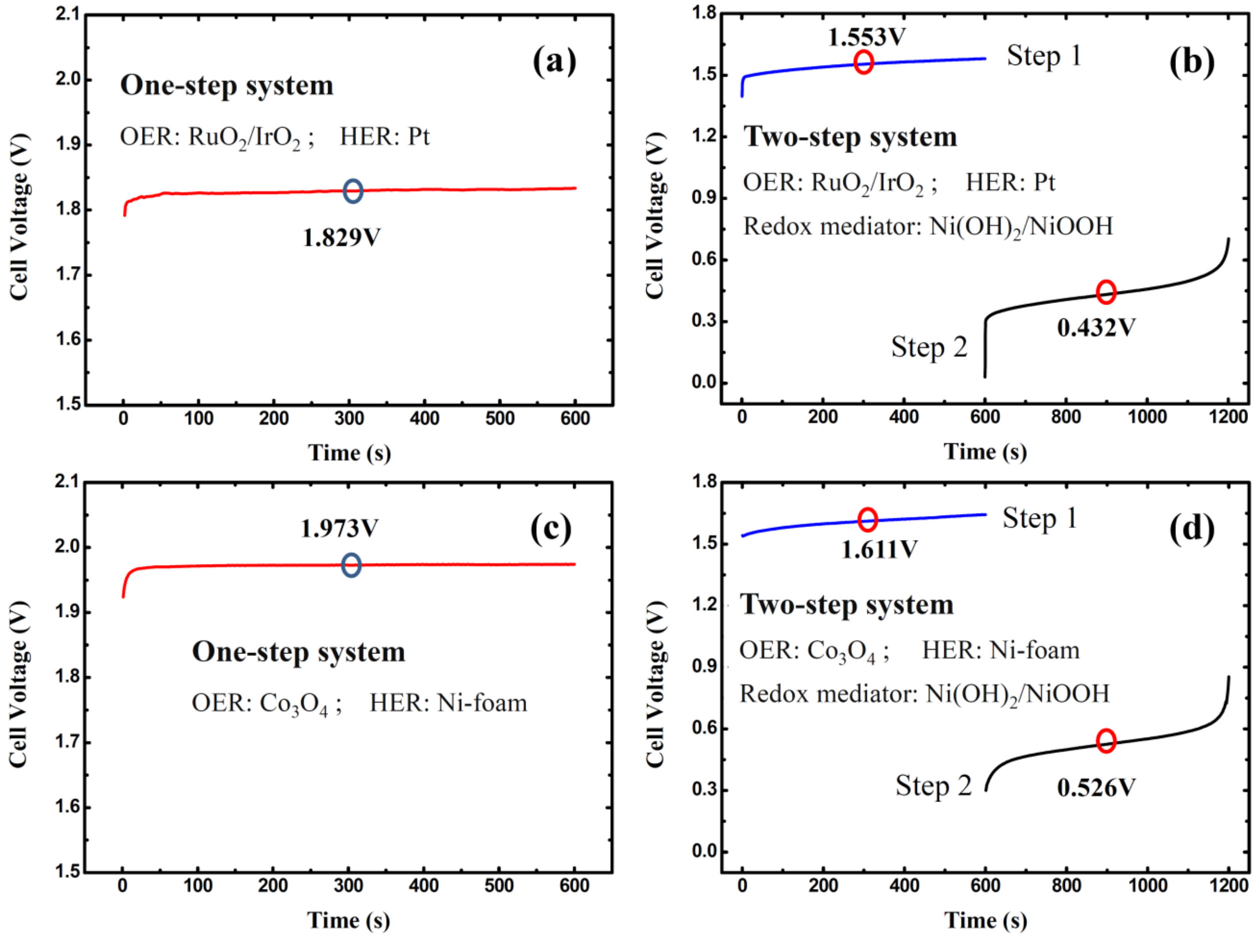

Supplementary Figure 14 | Driven voltage comparison at 200 mA between one-step system and two-step system. (a) one-step electrolysis using precious electrodes. (b) two-step electrolysis using precious electrodes. (c) one-step electrolysis using non-precious electrodes. (d) two-step electrolysis using non-precious electrodes.

In this experiment, chronopotentiometry measurement with an applied current of 200 mA was employed to investigate the one-step electrolysis process that is based on a commercialized RuO_2/IrO_2 coated Ti-mesh anode (2.5×4 cm^2) for OER and a commercialized Pt coated Ti-mesh cathode (2.5×4 cm^2) for HER in an alkaline medium. The achieved chronopotentiometry curve of one-step electrolysis is shown in Supplementary Figure 14a, where it can be detected that the cell exhibits a voltage of **1.829 V** with the applied current of 200 mA. The chronopotentiometry curve tested at 200 mA of two-step system using precious electrodes [RuO_2/IrO_2 coated Ti-mesh electrode (2.5×4 cm^2) , Pt coated Ti-mesh electrode (2.5×4 cm^2) and $Ni(OH)_2$ electrode (2.5×4 cm^2)] is shown Supplementary Figure 14b, where it can be observed that the two steps display a total cell voltage of **1.985 V** (1.553 + 0.432 V). Therefore, the efficiency of the two-step cell using precious electrodes should be **92%** (=1.829/1.985) compared to corresponding one-step system. In addition, the one-step system that is based on non-precious electrodes [Co_3O_4-based anode (2.5×4 cm^2) + Ni-foam cathode (2.5×4 cm^2)] was investigated by chronopotentiometry measurement with an applied current of 200 mA (Supplementary Figure 14c**).** It can be observed from Supplementary Figure 14c that the one-step system using non-precious electrodes exhibits a cell voltage of **1.973 V**. At the same test condition, the two-step system using non-precious electrodes displays a total cell voltage of **2.137 V** (1.611 + 0.526 V) (Supplementary Figure 14d). The efficiency also is about **92%** (=1.973/2.137) compared to corresponding one-step system.

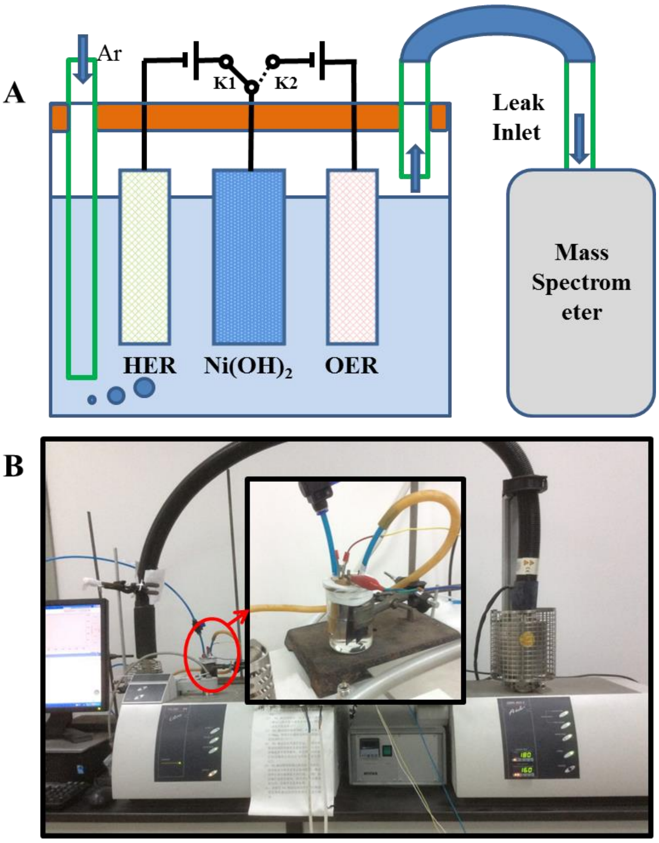

Supplementary Figure 15 | Schematic illustration and photo of the electrolysis cell connected to the mass spectrometer devices.

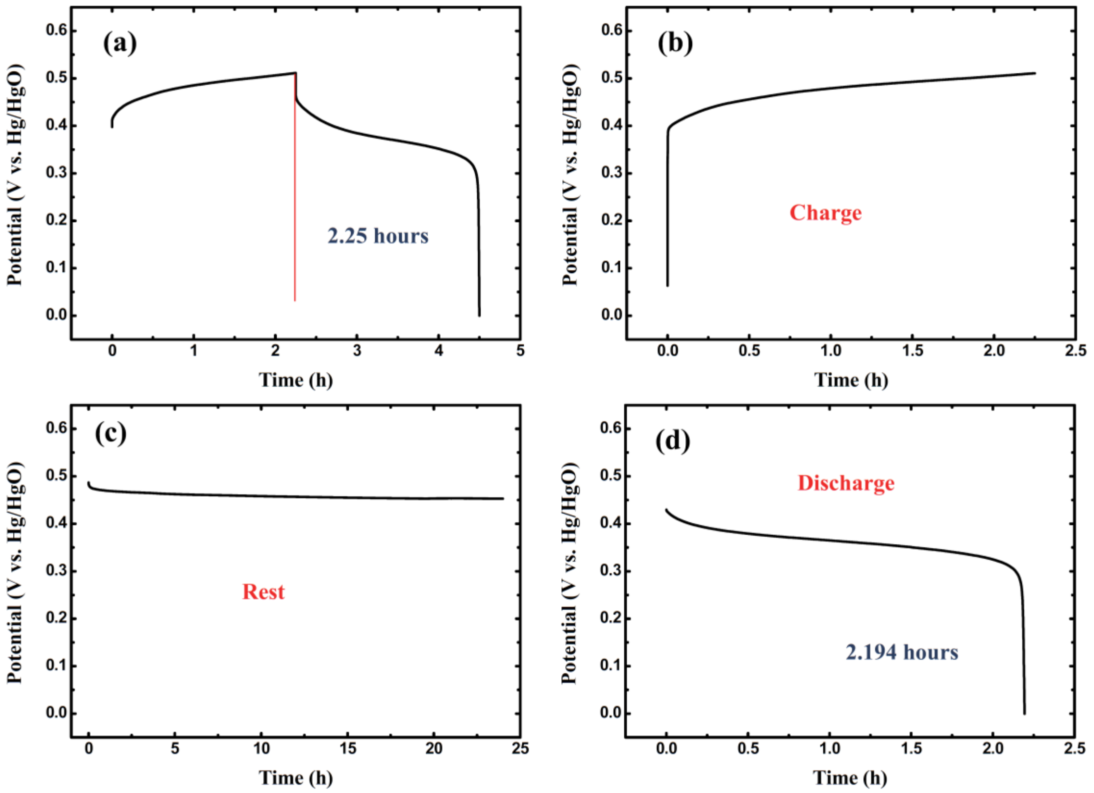

Supplementary Figure 16 | Self-discharge profile of charged $Ni(OH)_2$ electrode tested with three-electrode method [Work electrode: $Ni(OH)_2$ electrode; Counter electrode: Pt coated Ti-mesh (2.5×4 cm^2); Reference electrode: Hg/HgO; Electrolyte: 1 M KOH]. (a) Charge/discharge curve at a current of 100 mA of the electrode without rest [In this experiment, the $Ni(OH)_2$ was charged for 2.25 hours, and then was directly discharged to 0 V (vs. Hg/HgO)]. (b, c, d) the same $Ni(OH)_2$ electrode was cycled at the current of 100 mA with a consecutive three-step, including a charge step of 2.25 hours (b), a rest step of 24 hours (c) and a discharge step(d).

Self-discharge is a very common phenomenon for batteries. A very limited self-discharge is a quality index of any successfully commercialized battery. $Ni(OH)_2$-based rechargeable batteries (such as nickel metal hydride (Ni-MH) batteries and nickel–cadmium (Ni-Cd) batteries) have been commercialized for a very long time, and still play an important role on current battery market. Therefore, it is undoubted that the self-discharge of nickel hydroxide electrode is very limited. We believe that a lot of readers have the experience that their full charged Ni-MH or Ni-Cd batteries still can work even after several weeks (or months) rest. To clarify this point, self-discharge performance of a NiOOH electrode (2.5×4 cm^2) was investigated with three-electrode method. Firstly, the $Ni(OH)_2$ electrode was cycled with an applied current of 100 mA without any rest time between charge and discharge (Supplementary Figure 16a). In the experiment, the $Ni(OH)_2$ was charged for 2.25 hours, and then was directly discharged to 0 V (vs. Hg/HgO). As shown in Supplementary Figure 16a, the discharge time is 2.25 hours, indicating totally reversibility. Next, the same $Ni(OH)_2$ electrode was cycled at the current of 100 mA with a consecutive three-step, including a charge step of 2.25 hours (Supplementary Figure 16b), a rest step of 24 hours (Supplementary Figure 16c) and a discharge step (Supplementary Figure 16d). Supplementary Figure 16b shows the charge curve of the $Ni(OH)_2$ electrode over 2.25 hours. Supplementary Figure 16c shows the potential (vs. Hg/HgO) change of the charged $Ni(OH)_2$ electrode over rest of 24 hours, where it can be detected that the potential

reduces slightly. The discharge curve after 24 hours rest of the electrode is given in Supplementary Figure 16d. As shown in Supplementary Figure 16d, the electrode after 24 hours rest still can be discharged for 2.194 hours, which is close to the discharge time of the electrode without any rest (2.25 hours; see Supplementary Figure 16a), indicating a very limited self-discharge.

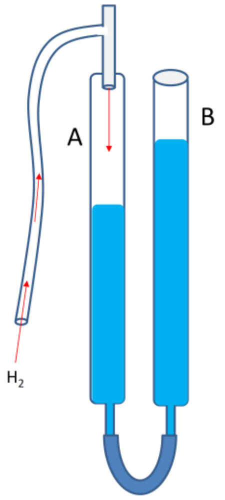

Supplementary Figure 17 | Schematic illustration of a typical drainage devices for gas volume measurement. [Photo of the device is given in Figure 3c] As shown in Supplementary Figure 17, the generated gas can be collected in tube-A. We should shift the tube-B during the measurement to keep the liquid level in tube-B be as same as that in tube-A, then we can read the volume of generated gas.

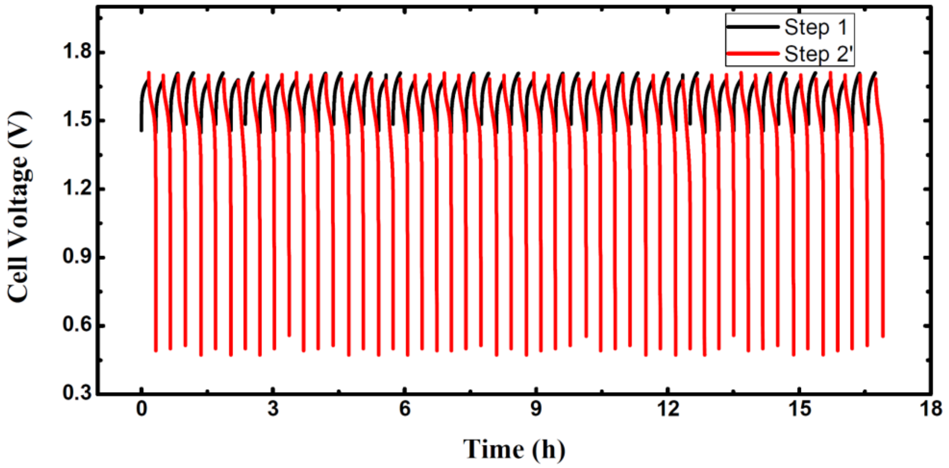

Supplementary Figure 18 | electrochemical profile of 50 cycles of H_2 production (step 1) and discharge of Zn-NiOOH battery (step 2′) [Test current: 200 mA; charge-time: 600 s; Cell structure: Pt coated Ti-mesh electrode (2.5×4 cm^2) for HER / Ni(OH)$_2$ electrode (2.5×4 cm^2) / Zn-anode (2.5×4 cm^2)]

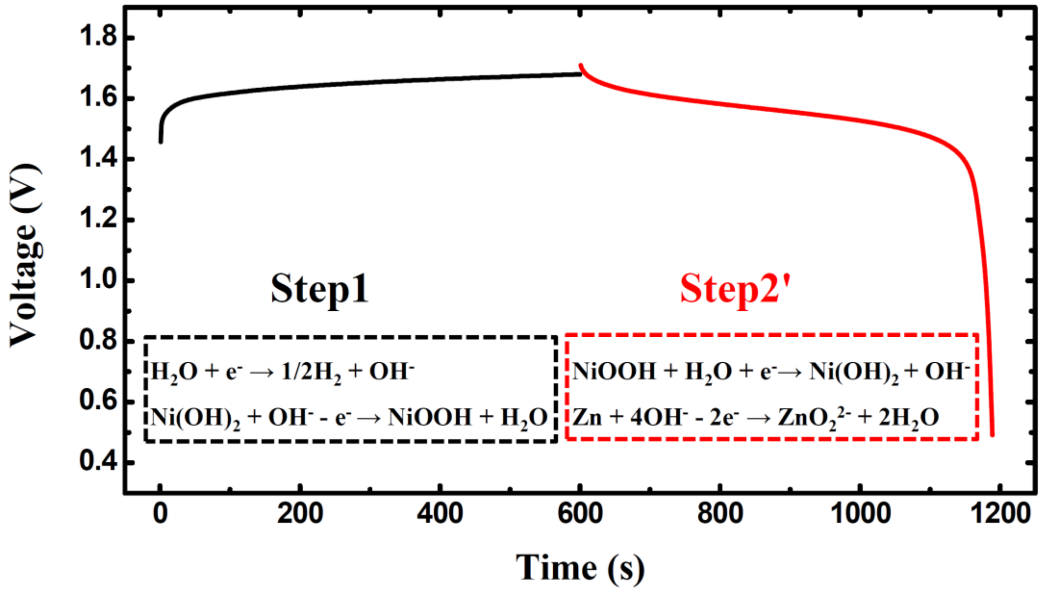

Supplementary Figure 19 | Charge curve of the H_2-production process (Step 1, black line) and the discharge curve of NiOOH-Zinc battery (Step 2′, red line). Inset: corresponding electrode reactions (charge current 200 mA; discharge current: 200 mA).

As shown in Supplementary Figure 19, the active materials for the charge/discharge cycle are H_2O, $Ni(OH)_2$ and Zn. According to these electrode reaction equations, it need 1 mol H_2O (18g), 1 mol $Ni(OH)_2$ (92.7g) and 0.5 mol Zn (0.5 × 65.38g) to store/deliver 1 mol electron (= 96500 C). Therefore, the energy density of the recharge cycle can be calculated by following equation

$$\mathrm{E} = \frac{Q \times V}{M_{Ni(OH)_2} + M_{H_2O} + 0.5M_{Zn}} \times \frac{1000}{3600}$$

Herein, $\boldsymbol{E}$ is the energy density (Wh kg^{-1}), **Q** is the quantity of electricity (96500C),$\boldsymbol{V}$ is the average discharge voltage of the cell (1.5V), $\boldsymbol{M_{Ni(OH)2}}$ is the molecular mass of $Ni(OH)_2$ (92.7g), $\boldsymbol{M_{H2O}}$ is the molecular mass of H_2O (18g) and $\boldsymbol{M_{Zn}}$ is the molecular mass of Zinc (65.38g). Thus, the calculated energy density can reach 280 Wh kg^{-1}. This theoretical energy density is close to the theoretical energy density of conventional Ni-MH batteries, Ni-Cd batteries or Ni-Zn batteries, and is higher than the theoretical energy density of lead-acid batteries and aqueous Li-ion batteries [2].

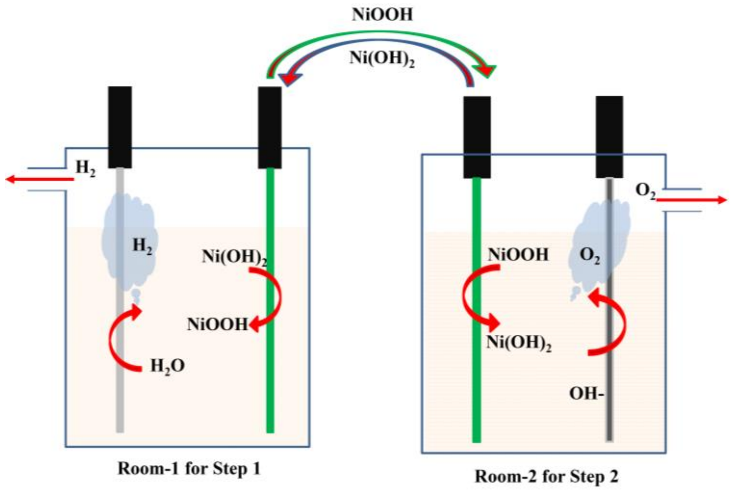

Supplementary Figure 20 | A schematic illustration of the two-step alkaline water electrolytic with two separate rooms for H_2 production (step 1) and O_2 production (step 2), respectively. Step 1 (H_2 production) takes place in room-1, which includes an anode reaction [$Ni(OH)_2 + OH^- - e^- \rightarrow NiOOH + H_2O$] and a cathode reaction [$H_2O + e^- \rightarrow 1/2H_2 + OH^-$]. Then, the formed NiOOH electrode in room-1 is moved to room-2 for Step 2, which includes an anode reaction [$2OH^- - 2e^- \rightarrow 1/2O_2 + H_2O$] and a cathode reaction [$NiOOH + H_2O + e^- \rightarrow Ni(OH)_2 + OH^-$].

As shown in Supplementary Figure 20, the anode reaction [$Ni(OH)_2 + OH^- - e^- \rightarrow NiOOH + H_2O$] on Step 1 in room-1 releases proton (H^+), which reacts with OH^- to form H_2O. At the same time, H_2O is reduced into H_2 on HER electrode (= cathode) with the reaction of [$H_2O + e^- \rightarrow 1/2H_2 + OH^-$] in room-1. Then, the formed NiOOH electrode is moved to room-2 for O_2 production (Step 2). On Step 2, the proton is stored in NiOOH electrode through the cathode reaction of [$NiOOH + H_2O + e^- \rightarrow Ni(OH)_2 + OH^-$], and simultaneously OH^- is oxidized on the anode (i.e. OER electrode) through the reaction of [$2OH^- - 2e^- \rightarrow 1/2O_2 + H_2O$]. As mentioned above, the nickel hydroxide ($Ni(OH)_2$/NiOOH) electrode is used as “a solid-state proton buffer” that can be moved between room-1 for H_2 production (step 1) and room-2 for O_2 production (step 2).

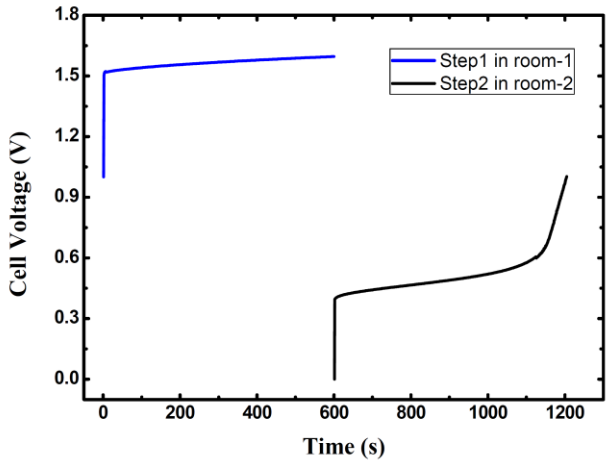

Supplementary Figure 21 | Chronopotentiometry curve (cell voltage vs. time) of the alkaline electrolytic cell with separate H_2/O_2 production in different rooms. In this experiment, step 1 is performed in room-1 with a current of 200 mA for 600s. Then, the formed NiOOH electrode is moved to room-2 for O_2 production with the same current of 200 mA. Please also see Movie S6 and S7. [Electrodes: Pt coated Ti-mesh electrode (2.5×4 cm^2) for HER / Ni(OH)$_2$ electrode (2.5×4 cm^2) / RuO_2/IrO_2 coated Ti-mesh electrode (2.5×4 cm^2) for OER]

In order to further clarify the operation model shown in Supplementary Figure 20, the H_2/O_2 production with two separate rooms was also investigated (see Supplementary Figure 21). The achieved electrochemical profile is as same as that achieved by one-compartment water electrolytic cell. In addition, Supplementary Movie 6 and 7 were given in revised manuscript to show the separate H_2/O_2 production with two separate rooms.

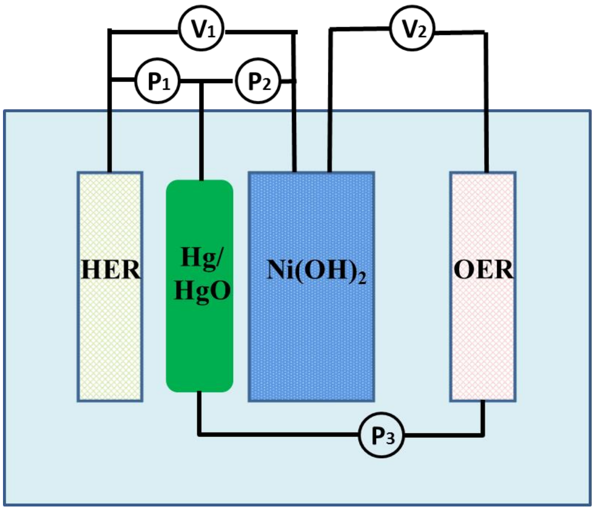

Supplementary Figure 22 | Schematic illustration of the voltage (or potential) record for water electrolysis investigating.

Supplementary References:

1. Zeng, K., Zhang, D. K. Recent progress in alkaline water electrolysis for hydrogen production and applications. *Progress in Energy and Combustion Science* **36**, 307-326 (2010).
2. Luo, J. Y., Cui, W. J., He, P., Xia, Y. Y. Raising the cycling stability of aqueous lithium-ion batteries by eliminating oxygen in the electrolyte. *Nat. Chem.* **2**, 760-765 (2010).
